# Maternal death surveillance and Primary Health Care nurses’ performance in a Brazilian capital city*

**DOI:** 10.1590/1518-8345.7898.4948

**Published:** 2026-10-16

**Authors:** Núria Ananda Parron Giacomelli Pereira, Shaiane de Farias Durval, Albert Schiaveto de Souza, Elen Ferraz Teston, Sonia Silva Marcon, Maria de Lourdes de Souza, Bianca Cristina Ciccone Giacon-Arruda

**Affiliations:** 1 Universidade Federal do Mato Grosso do Sul, Instituto Integrado de Saúde, Campo Grande, MS, Brazil.; 2 Universidade Federal do Mato Grosso do Sul, Instituto de Biociências, Campo Grande, MS, Brazil.; 3 Universidade Estadual de Maringá, Departamento de Enfermagem, Maringá, PR, Brazil.; 4 Universidade Federal de Santa Catarina, Florianópolis, SC, Brazil.

**Keywords:** Maternal Mortality, Maternal Death, Public Health Surveillance, Primary Health Care, Family Health, Nursing

## Abstract

**Objective:**

to identify the factors related to Primary Health Care nurses’ performance in maternal death surveillance.

**Method:**

an epidemiological and descriptive-analytical study conducted with 143 nurses from a capital city in the Brazilian Midwest region. The data were collected by means of a structured and self-applied questionnaire and analyzed through descriptive and inferential statistics using the Chi-square test and via a multivariate analysis resorting to binary logistic regression, considering a 5% significance level.

**Results:**

most of the participants had been working in their current job position for less than five years and had not undergone any training in maternal death surveillance; however, they were aware about its objective and about the Municipal Maternal Mortality Committee (and its goals). The participants’ time in their current job position was associated with participation in training, with conducting investigation procedures, with knowing about the committee and with taking part in its meetings. Training was associated with knowing about the investigation objective, the committee, its goals and participation in meetings. The nurses that had attended training presented an Odds Ratio of 5.82 (95%CI: 2.46-13.75) as for already having conducted a maternal death investigation process.

**Conclusion:**

permanent education is a predictive factor for nurses’ performance and should be enhanced by institutional policies.

## Highlights

**(1)** Maternal death surveillance is an essential action in Primary Health Care.

**(2)** Nurses play a crucial role in investigating maternal deaths.

**(3)** Training and knowing about the Maternal Mortality Committee exert an influence on the process.

**(4)** It is fundamental to discuss maternal death cases in team meetings.

**(5)** It is indispensable to reinstate Maternal Mortality Committee analyses to Primary Health Care.

## Introduction

Maternal Death (MD) is considered one of the main Public Health challenges at the global level. Target 3.1 from the Sustainable Development Goals (SDGs) sets the objective of reducing the Maternal Mortality Ratio (MMR) to less than 70 deaths for every 100,000 live births by 2030. Despite this international commitment, the MMR was estimated at 223 deaths for every 100,000 live births in 2020, which evidences the need to intensify efforts to attain the intended target^1^.

In Brazil, the MMR rose from 57.9 in 2019 to 74.7 in 2021, reversing the decreasing trend observed since 1990. These values are distant from the national target, which foresees reaching an MMR of 30 by 2030^2^. The main causes of Maternal Mortality (MM) are as follows: obstetric hemorrhage; hypertensive disorders; and indirect causes (pre-existing conditions generally exacerbated by pregnancy). Most of these deaths can be avoided and are related both to individual factors (race/skin color, income, schooling and area of residence) and to contextual ones (inequalities in access and in health care quality)^3^ ^)-(^ ^4^.

Given this context, the World Health Organization (WHO) recommends implementing Maternal and Perinatal Death Surveillance and Response (MPDSR) as a strategy to reduce the number of avoidable deaths and understand the social determinants related to maternal health^5^ ^)-(^ ^6^. In Brazil, it is mandatory to investigate deaths of women of reproductive age; such investigation should be in charge of health professionals with the support of maternal mortality committees, which gather different institutional and civil society actors to analyze cases and propose improvements^7^ ^)-(^ ^8^.

However, the effective implementation of MPDSR still faces challenges, especially in low- and middle-income countries and including difficulties related to availability of duly trained professionals, infrastructure, funding and data quality^9^ ^)-(^ ^10^. It is estimated that 34% of all maternal death records are incomplete and that up to 39% of the deaths are underestimated due to under-reporting or to inadequate classification^11^. In Brazil, an investigation conducted with 88% of the 64,000 deaths of women of reproductive age in 2019 resulted in a 32.7% increase in the number of officially recognized maternal deaths^12^.

In the Brazilian health model, PHC nurses are responsible for activities that encompass from assistance in the pregnancy-puerperal cycle (pre-natal assistance, childcare, risk assessment, active search) to implementing educational and death investigation actions^13^ ^)-(^ ^14^. In this scenario, there is an evident need to qualify and improve maternal death surveillance in its different aspects and spaces. Primary Health Care (PHC) and its professionals (especially nurses) are fundamental in this process^15^.

Given the above and considering nurses’ central role in the territory and in leading health teams, it is essential to understand the factors that exert an influence on these professionals’ participation in MPDSR to qualify prevention strategies for avoidable maternal deaths and to improve public policies and maternal health programs. This will contribute to reducing the scope of a problem that affects women’s fundamental rights, such as the right to sexual and reproductive health and access to suitable health services during the pregnancy-puerperal cycle^14^ ^),(^ ^16^.

Thus, the objective of this study was to identify the factors associated with PHC nurses’ performance in maternal death surveillance.

## Method

### Type of study

An epidemiological and descriptive-analytical study. The recommendations set forth in the Strengthening the Reporting of Observational Studies in Epidemiology (STROBE) statement^17^ were followed to structure and organize it.

### Locus

The study was conducted at a number of health units belonging to the PHC level, that is, the gateway to the Brazilian Unified Health System (*Sistema Único de Saúde*, SUS), attached to the Municipal Health Department from a capital city in the Midwest region of the country. During the study period, the aforementioned capital city had 14 Basic Health Units (BHUs), 54 Family Health Units (FHUs) and three Family Clinics (FCs), totaling 71 units distributed across seven health regions. Thus, the Municipal Committee for the Prevention of Maternal, Child and Fetal Mortality (*Comitê Municipal de Prevenção de Mortalidade Materna, Infantil e Fetal*, CMPMMIF) was created in 2001.

### Period

The data collection period encompassed from December 2019 to March 2021, facing the organizational and epidemiological issues imposed by the COVID-19 pandemic.

### Population

The study population was comprised by 204 nurses linked to the 71 participating health units. The decision to include these professionals was due to the fact that, in the municipality researched and as recommended by the local surveillance protocols, PHC nurses are responsible for conducting home visits and interviews with the families to investigate deaths of women of reproductive age, such as maternal death cases.

### Selection criteria

The inclusion criteria were as follows: being nurses, of both genders and regardless of the type of employment contract (permanent or fixed-term). No restrictions were imposed as for working time or experience related to maternal death investigation. The professionals excluded were those that were away from the activities developed in the health units (regardless of the reason) or on vacation at the data collection time. In turn, incomplete questionnaires (no answers in at least 10% of the data collection instrument questions) were excluded.

### Definition of the sample

The sample was recruited for convenience with the purpose of encompassing the entire municipal territory, a relevant strategy for research studies about work processes in PHC. However, as data collection coincided with the COVID-19 pandemic period (which may have affected the professionals’ participation), it was decided to perform the classical minimum sample size calculation to estimate proportions in finite populations, aiming at supporting interpretation of the results. The result indicated a minimum sample size of 103 participants to ensure statistical precision, considering 204 eligible nurses, a 95% confidence level and a 5% maximum tolerable error^18^.

All the eligible nurses were invited to take part in the study; six were not included for being away from their work activities at the data collection time and 55 failed to return the questionnaire without providing any reason, even after at least three contact attempts and reminders sent by the lead researcher. The final sample was comprised by 143 professionals; the number of participants was higher than the one calculated for the minimum sample to ensure analytical soundness^18^. In addition, the rates of responding nurses for each of the seven health regions were above 60%, as shown in Table 1.

**Table 1:** Number of nurses allocated to PHC* and of research participants, in addition to the respective percentages according to health regions. Campo Grande, MS, Brazil, 2019

Health regions	Number of nurses	Number of participating nurses	%^†^ of participating nurses
Region 1	55	41	74.5
Region 2	36	26	72.2
Region 3	36	23	63.9
Region 4	29	20	69
Region 5	28	18	64.3
Region 6	13	8	61.5
Region 7	7	7	100
Total	204	143	70.1

*PHC = Primary Health Care; ^†^% = Percentage

### Study variables

The variables used to describe nurses’ performance in maternal death surveillance at the PHC level were as follows: having conducted maternal death investigation procedures as nurses (Yes/No); first information source about the death (Family member/Community Health Agent [CHA]/Health surveillance technician/Others; nurses providing pre-natal assistance to women (Yes/No); modality of the first contact with the family for the investigation (CHA/Phone call/Home visit/Other); presence of another professional during the home visit for the investigation (Yes/No); family refusal to take part in the investigation (Yes/No); support provided by the surveillance team (Yes/No); difficulties filling in the Maternal Death Investigation Form - Outpatient Health Service (Yes/No) and the Maternal Death Investigation Form - Home Interview^19^ (Yes/No); having been provided with recommendations based on the analysis made by the Maternal Mortality Committee (Yes/No); and discussing cases in team meetings (Yes/No).

The variables related to the professionals’ training and knowledge were the following: having undergone training in maternal death surveillance (Yes/No); knowing about the investigation objective (Yes/No); knowing about the existence of the municipal MM committee (Yes/No) and about its objective (Yes/No), and participating in its meetings; in addition to time in the current job position (in full years).

The following variables were considered for the sociodemographic, professional and training characterization: gender (Female/Male); age (years); type of health unit currently allocated to (BHU/FHU/FC); undergraduate Nursing training time (in years); complementary training (Yes/No/Omitted) and level (*lato sensu*, *stricto sensu*, Both or Not reported).

### Instruments used to collect the information

The data were collected by means of a structured and self-applied questionnaire prepared by the lead researcher. For such purpose, the first step was to map the recommendations from the Ministry of Health and those found in the literature on maternal death surveillance, in order to define the dimensions to be investigated. The first version of the instrument was presented to and discussed with a researcher experienced in studying maternal death aiming at assessing clarity, pertinence and scope of the items, suggesting minor changes in how to present some questions and answer options.

Subsequently, a PHC nurse with previous experience in maternal death investigation procedures was asked to evaluate and answer the questionnaire: she only suggested changes in the order of the questions. The final version of the instrument consisted in 34 structured questions, organized into three blocks: 1) Sociodemographic, work and professional training characteristics (eight questions); 2) Knowledge and training about maternal death investigation processes and the MM committee (five questions); and 3) The participants’ professional experience in conducting investigation procedures (21 questions). In this study, the variables used to describe nurses’ performance in maternal death surveillance at the PHC level were part of this instrument and have been described in the previous item.

### Data collection

For data collection purposes, the lead researcher requested authorization to announce and initiate the research study to the coordinating offices of the seven health regions from the municipality. In order to standardize the application process, the lead researcher herself handed in an envelope containing the data collection instrument and guidelines on how to fill it in and return it, in addition to two copies of a Free and Informed Consent Form (FICF). At that moment, the same guidelines were provided orally to all participants to reduce possible collection biases.

Along with the signed FICFs, the completed instruments would be placed in the same envelopes, sealed and delivered to the health unit manager, who was already instructed about forwarding them to the respective coordinating office of the health region for the researcher to collect them. Nevertheless, periodic contacts with the units and the professionals were necessary to reinforce reminders about participating in the study and answering the instrument. Consequently, the questionnaires were collected once a month, both in the regional coordinating offices and directly at the health units.

Some participants asked for a new envelope with the instrument for having lost the material or for having entered the team after the collection period had started. This situation was mainly due to the public selection process implemented, with the consequent substitution of temporary professionals for permanent employees, as well as to the COVID-19 pandemic, which required reorganizing the assistance provided and re-scheduling the professionals’ activities among different services.

### Data treatment and analysis

The answers given to the questionnaires were double-typed into a Microsoft Excel 2013 spreadsheet to identify possible inconsistencies and analyzed in the Jamovi^®^ statistical software program, adopting a 5% significance level. The Chi-square test was employed to verify the association between training and what nurses knew about maternal death surveillance. A binary logistic regression was performed to identify factors associated with conducting maternal death investigation procedures (outcome), estimating adjusted Odds Ratios (ORs) and 95% Confidence Intervals (95%CIs)^20^.

The variables were prepared by means of coding schemes and categorization, recoding some to adapt them to the test used: age in age groups (18-30, 31-40, 41-50, 51-60 and 61+ years old); training time and working time dichotomized based on the median, with a value of six years (lower than/higher than or equal to the median); complementary training (Yes/No) and level (Undergraduate degree/Graduate degree); and modality of the first contact with the family for the investigation (In-person - CHA and home/remote visit - Phone call and Others).

Therefore and respecting absence of multicollinearity (examined with the Variance Inflation Factor and by analyzing tolerance), all the independent variables were included in the initial model, which was subsequently refined using the “backward stepwise” method to reach the final model. Fit quality was assessed by means of global statistical values (deviance and Akaike and Bayesian information criteria), pseudo-R² (Cox & Snell and Nagelkerke) and the likelihood ratio test^21^.

In addition, in order to assess soundness and discriminatory capacity, a predictive performance analysis was performed on the final binary regression model. The global accuracy, sensitivity, specificity, area under the ROC curve (AUC) and Brier score metrics were estimated, which allowed examining stability of the model, complementing the global fit assessment, given the organizational and multifactorial nature of the outcome^19^.

### Ethical aspects

The study was approved by the Committee of Ethics in Research with Human Beings of the signatory institution, as per CAAE 22855519.1.0000.0021.

## Results

Most of the 143 nurses that took part in the study belonged to the female gender (n=130; 90.9%), were aged between 18 and 60 years old and had been working in their current job position for less than six full years (n=101; 71.6%). As for allocation units, 74.8% were allocated to FHUs (n=107), 17.5% to BHUs (n=25) and 7.7% to FCs (n=11). In relation to training, 65.8% (n=94) had up to 10 years of undergraduate Nursing studies and 79.6% (n=110) reported complementary training at the graduate level, predominantly in the *lato sensu* modality (n=103; 72%).

Regarding maternal death investigation, 58.7% (n=84) reported having already conducted one such procedure, not observing statistically significant differences as for type of unit and having undergone training (p=0,396), conducting maternal death investigation procedures (p=0.942) or in terms of discussing cases in team meetings (p=0.297). The main information source about the deaths were CHAs (n=34; 41.5%), followed by health surveillance technicians (n=24; 29.3%) and by family members (n=18; 22%).

Most of the nurses were not responsible for the dead pregnant women’s pre-natal assistance (n=59; 70.2%). The most usual modality of the first contact with the families for the investigation was home visit (n=42; 52.5%), frequently along with another professional from the team (n=62; 76.5%). As for institutional support, 53.6% (n=45) reported support from the surveillance team; in turn, 46.4% (n=39) indicated absence of support.

Difficulties filling in the investigation forms were pointed out by 11% of the participants, and only one nurse (1.2%) reported having received recommendations from the Maternal Mortality Committee after analyzing the death. Discussion of the cases in team meetings was mentioned by 32.9% (n=27) of the professionals, and 30.5% (n=25) reported that the families refused to take part in the home-based investigation.

Although only 34.3% (n=49) of the nurses had undergone specific training in maternal death surveillance, most of them reported knowing the investigation goal (n=121; 84.6%), as well as being aware about the existence of a Maternal Mortality Committee in the municipality (n=100; 65.9%) and about its objective (n=100; 69.9%). However, only 17.5% (n=25) attended the committee meetings.

The time in the current job position variable presented a significant association with having undergone training in maternal death surveillance (p<0.001), with knowing about the existence of the Maternal Mortality Committee (p<0.001) and with attending the Maternal Mortality Committee meetings (p=0.005) (Table 2).

**Table 2:** Association between study variables and time in the current job position (n* = 143). Campo Grande, MS, Brazil, 2019-2021

Variables	Time in the current job position (PHC^†^)		
	<6 years	6+ years	p-value^‡^
	n* (%)^§^	n* (%)^§^	
Training in maternal death surveillance			
Yes	21 (20.8)	28 (70)	<0.001
No	80 (79.2)	12 (30)	
Knowledge of the Municipal Maternal Mortality Committee			
Yes	62 (62.6)	37 (92.5)	<0.001
No	37 (37.4)	3 (7.5)	
Knowledge of the Municipal Maternal Mortality Committee objective			
Yes	64 (66)	35 (89.7)	0.005
No	9 (29.0)	24 (36.4)	
Participation in Committee meetings			
Yes	4 (12.1)	13 (32.5)	0.004
No	89 (88.1)	27 (67.5)	
Knowledge of the maternal death investigation objective			
Yes	84 (85.7)	36 (92.3)	0.291
No	14 (14.3)	3 (7.7)	

*n = Size of the sample comprised by nurses; ^†^PHC = Primary Health Care; ^‡^Chi-square test, considering a 5% significance level; ^§^% = Percentage

Having undergone training in maternal death surveillance presented significant associations with the variables related to knowing about the death investigation objective (p=0.025) and about the existence of the Maternal Mortality Committee (p<0.001) and its goal (p<0.001), as well as with attending some committee meeting (p=0.003), as shown in Table 3.

**Table 3:** Association between study variables and training in maternal death surveillance (n* = 143). Campo Grande, MS, Brazil, 2019-2021

Variables	Training in maternal death surveillance		
	Yes	No	p-value^‡^
	n* (%^†^)	n* (%^†^)	
Knowledge of the Municipal Maternal Mortality Committee			
Yes	46 (93.9)	54 (58.7)	<0.001
No	3 (6.1)	38 (41.3)	
Knowledge of the Municipal Maternal Mortality Committee objective			
Yes	45 (93.8)	55 (61.1)	<0.001
No	3 (6.3)	35 (38.9)	
Participation in committee meetings			
Yes	15 (30.6)	10 (10.6)	0.003
No	34 (69.4)	84 (89.4)	
Knowledge of the maternal death investigation objective			
Yes	46 (95.8)	75 (82.4)	0.025
No	2 (4.2)	16 (17.6)	
Difficulty in completing the Maternal Death Investigation Form - M1^§^			
Yes	2 (5.0)	7 (16.7)	0.091
No	38 (95.0)	35 (83.3)	
Difficulty in completing the Maternal Death Investigation Form - M3^||^			
Yes	3 (7.5)	6 (14.3)	0.326
No	37 (92.5)	36 (85.7)	

*n = Size of the sample comprised by nurses; ^†^% = Percentage; ^‡^Chi-square test, considering a 5% significance level; ^§^M1 = Maternal Death Investigation Form - Outpatient Health Service; ^||^M3 = Maternal Death Investigation Form - Home Interview

In turn, knowing about the existence of the Maternal Mortality Committee was significantly associated with knowing about its goal, attending its meetings and greater knowledge about the objective of maternal death investigation procedures (Table 4).

**Table 4:** Association between study variables and knowing about the existence of a Maternal Mortality Committee in the municipality (n* = 143). Campo Grande, MS, Brazil, 2019-2021

Variables	Knows about the Municipal Maternal Mortality Committee		
	Yes	No	p-value^‡^
	n* (%^†^)	n* (%^†^)	
Knowledge of the Municipal Maternal Mortality Committee objective			
Yes	84 (87.5)	14 (35.0)	<0.001
No	12 (12.5)	26 (65.0)	
Participation in committee meetings			
Yes	24 (24.0)	1 (2.4)	0.002
No	76 (76.0)	40 (97.6)	
Knowledge of the maternal death investigation objective			
Yes	90 (92.8)	29 (72.5)	0.001
No	7 (7.2)	11 (27.5)	

*n = Size of the sample comprised by nurses; ^†^% = Percentage; ^‡^Chi-square test, considering a 5% significance level

Table 5 presents the coefficients corresponding to the final binary logistic regression model. It can be seen that the intercept was not statistically significant (β=-0.128; SE=0.207; Z=-0.618; p=0.536). On the other hand, having undergone training proved to be strongly associated with conducting maternal death investigation procedures, with a significant positive coefficient (β=1.762; SE=0.438; Z=4.020; p<0.001). This estimate corresponds to an Odds Ratio of 5.82 (95%CI: 2.46-13.75), indicating that the professionals that had undergone training were almost six times more likely to already having conducted some investigation process when compared to those not duly trained.

**Table 5:** Coefficients corresponding to the final binary logistic regression model between professional/operational variables and Primary Health Care nurses having already conducted maternal death investigation procedures. Campo Grande, MS, Brazil, 2019-2021

Predictors		β^*^	SE^†^	Z^‡^	p^§^	OR^||^ (95%CI^¶^)
Intercept		-0.128	0.207	-0.618	0.536	0.88 (0.58 - 1.32)
Training in maternal death surveillance						
	Yes - No	1.762	0.438	4.020	<0.001	5.82 (2.46 - 13.75)
Deviance=174; AIC^**^=178; R²CS^††^=0.128; R²N^‡‡^=0.173; VIF^§§^=1.00; Tolerance=1.00						

*β = Estimated coefficient in the logistic model (logit); ^†^SE = Standard Error of the estimate; ^‡^Z = Wald’s statistical test; ^§^p = Significance level; ^||^OR= Odds Ratio); ^¶^95%CI = 95% Confidence Interval; **AIC = Akaike Information Criterion; ^††^R²CS = Cox & Snell coefficient of determination; ^‡‡^R²N = Nagelkerke coefficient of determination; ^§§^VIF = Variance Inflation Factor

The logistic regression model presented satisfactory global fit, evidenced by a deviance value of 174 and by the Akaike Information Criterion (AIC=178), suggesting good parsimony between complexity and predictive capacity. The pseudo-R² measures indicated moderate performance of the model, with Cox & Snell R² equal to 0.128 and corrected Nagelkerke R² of 0.173. No collinearity was evidenced after evaluating the model assumptions, as the Variance Inflation Factor (VIF=1.00) and the tolerance value (1.00) indicated total independence between the predictor included and absence of structural redundancy that might compromise stability of the estimates.

The predictive performance analysis indicated 63.6% accuracy, with an area under the ROC curve (AUC) of 0.672, suggesting moderate discriminatory capacity. The model presented high specificity (86.2%) and moderate sensitivity (48.2%), indicating better capacity to properly identify professionals that had not conducted maternal death investigation procedures. The Brier Score was 0.210, evidencing an acceptable calibration error and stability of the estimated odds.

## Discussion

The results obtained in this study indicated that PHC nurses’ performance in maternal death surveillance is directly related to having undergone specific training on the topic, emerging as the only factor significantly associated with having already conducted maternal death investigation procedures. With almost six times higher chances among trained professionals, the magnitude of this association reinforces centrality in permanent education as a structuring element to strengthen health surveillance and to qualify research practices in the PHC scope^22^.

Although most of the participating nurses had complementary training at the *lato sensu* graduate level, a low percentage of professionals undergoing specific training programs on maternal death surveillance was noticed. In addition, the absence of differences across the types of performance units indicates that the weaknesses related to discussing cases and to maternal death investigation procedures are not restricted to a single specific service model, but reflect organizational challenges that permeate primary care.

This finding suggests that, even if advanced, generalist academic training does not suffice to ensure the development of the technical, analytical and operational competencies required for properly conducting death investigation processes, corroborating diverse evidence which shows that maternal mortality surveillance demands specific and updated knowledge in line with the national and international guidelines^23^.

In this context, permanent education contributes to improving technical autonomy and professional confidence, favoring access to national and international guidelines and the incorporation of practices in line with public policies targeted at reducing the maternal mortality ratio. Although absence of training does not indicate unawareness regarding the maternal death surveillance principles, the results suggest that the necessary expertise to conduct investigation procedures is not exclusively built on practice time or on academic training but requires immersion into specific training processes targeted at surveillance and at qualifying the care provided^22^ ^)-(^ ^23^.

Having undergone training proved to be associated with longer working time in PHC, with knowing about the existence and objectives of the mortality committee and with attending meetings held by this body. These results indicate that working time presupposes accumulated experience, which can expand the professionals’ exposure to training and organizational processes related to surveillance and favor the development of specific competencies. However, the findings show that service time alone does not ensure effective participation in maternal death surveillance, reinforcing the need for systematic institutional strategies in the permanent education scope.

Specific training enables getting closer to the essential stages of the death surveillance process, enhancing collection and acquisition of important information to critically analyze each case in other network instances, such as MM committees and health surveillance teams^24^. The quality of the data recorded at each care network point and made available by health information systems is directly related to under-reporting and under-recording^25^, which hinders devising recommendations and the preventive potential of surveillance.

The logistic regression model presented moderate explanatory performance, as indicated by the Cox & Snell and Nagelkerke R² values, which reflects the multifactorial and organizational nature of maternal death surveillance and evidences that, although professional training emerges as a central factor, there are other relevant determinants not contemplated in the variables analyzed.

Institutional, organizational and contextual factors such as availability of human resources, institutional support, turnover of professionals, workloads, organizational culture and the workers’ mental health conditions can exert a significant influence on how investigation procedures are conducted and should be considered in future studies. Consequently, although training has been highlighted as a central factor, it should not be understood in an isolated way but integrated to a broader set of systemic interventions^23^.

The role of CHAs as first information source about maternal death stands out among the operational findings, as well as resorting to home visits as the predominant strategy for initial contacts with the families, frequently with the participation of more than one professional from the team. These practices are in line with the PHC and Health Surveillance principles, especially in terms of establishing a previous relationship with the pregnant women’s families, of early identifying events and of the interprofessional approach^18^ ^,^ ^26^. For being immersed in the community and due to their previous relationship with the families, CHAs play a strategic role in detecting and notifying deaths, reinforcing the importance of their training and articulation with nurses’ performance, who act as team coordinators^27^.

PHC can be implemented by means of different care models and organizational arrangements as long as they preserve essential attributes inherent to PHC as first contact and care longitudinality, integrality and coordination^25^. In the municipality under study, although PHC consists of health sectors comprised by different types of units (FHUs, BHUs and FCs), all of them are responsible for investigating maternal deaths in their attached territory, in line with the result obtained in this study, which did not indicate any statistically significant differences as for type of performance unit and having undergone training, conducting maternal death investigation procedures and discussing cases in team meetings.

Despite these potentialities, the study revealed critical operational weaknesses in the maternal death surveillance process. The fact that only one nurse was provided with recommendations from the Maternal Mortality Committee after analyzing a death case is particularly worrisome, as it impairs the pedagogical, preventive and transforming nature of surveillance. The absence of systematic assessments weakens feedback to the services, limits the incorporation of institutional lessons learned and reduces the potential of the recommendations to reorganize work processes and improve care quality^24^ ^)-(^ ^25^.

Similarly, the low percentage of professionals that reported discussing maternal death cases in team meetings evidences the weakness of the collective spaces for reflection and critical analysis of the assistance provided. Not holding these discussions hinders identifying avoidable assistance-related failures, impairs shared decision-making and weakens organizational learning^28^.

In the international scope, factors such as lack of time, institutional leadership and support, limited communication, scarcely clear professional roles and restricted resources also tend to hinder interprofessional collaboration in health services^29^ ^)-(^ ^32^. This scenario can be related to work overload, to lack of time devoted to meetings, to insufficiently valuing these practices in the PHC routine and to the weakness of an organizational culture targeted at the analysis of adverse events^33^.

In addition, assistance-related overload and the professionals’ physical wear out (intensified in the pandemic context) should also be considered as factors that may have exerted a negative influence on engaging in surveillance, permanent education and team discussion activities. Burnout and psychological distress among health professionals have been widely described in the literature as elements that affect care quality and adherence to institutional practices and should also be incorporated to the analysis about maternal death surveillance^25^ ^),(^ ^34^ ^)-(^ ^35^.

Another relevant finding refers to institutional support, as almost half of the nurses taking part in the study reported not receiving it from the health surveillance team. This gap evidences weaknesses in the articulation between PHC and the other surveillance system components, with the possibility of generating professional insecurity, operational difficulties and discontinuity in the investigative actions. Technical and institutional support is fundamental to ensure quality of the investigation procedures, especially in contexts marked by deeper social vulnerability and care complexity^25^.

The fact that a significant percentage of the investigating nurses were not involved in pre-natal follow-up also deserves to be noted. The pre-natal period is a fundamental moment to early identify obstetric risks, perform timely interventions and strengthen the relationship with each pregnant woman. Failures in this follow-up can reflect organizational problems, access-related limitations and insufficient resources, as well as social and cultural factors that exert an impact on women’s resorting to health services^36^ ^)-(^ ^37^. Family resistance or refusal to take part in home-based investigation procedures can be related to how they perceived the quality of the assistance provided and to each woman’s path in the care network.

However, these results should be interpreted considering the onset of the COVID-19 pandemic in the Brazilian context. Not controllable in the study planning phase, this event permeated almost the entire data collection process and triggered transformations in PHC operations. Among other changes, reorganization of the services, prioritization of emergency-related actions, suspension or reduction of assistance and organizational collective activities and limitations in terms of home visits may have contributed to the low adherence to the surveillance process stages noticed in the study, mainly in the permanent education and team discussion processes and in the institutional reinstatement of the committees’ analyses.

The pandemic context should also be considered in relation to the limitations of this study. Combined with the convenience sample and with not knowing data about the non-respondents, the non-response rate of approximately 30% among the eligible professionals (possibly related to care overload during this period) can introduce a response bias, as the professionals most overloaded or least involved in surveillance may have been less willing to participate, possibly overestimating the knowledge and engagement levels observed.

Added to the fact that the study was conducted in a single municipality and to the moderate explanatory performance of the model, these limitations point to the need for future research studies that include organizational, institutional and psychosocial variables, as well as analyses stratified by type of health unit, considering the specificities of the different PHC organization models.

Despite these limitations, the study managed to encompass professionals from all health regions of the municipality and included more participants than the minimum sample to ensure analytical soundness. Even if not representative due to sample selection biases, the findings consistently evidence^18^ modifiable factors that can support managers from different contexts in formulating institutional policies targeted at strengthening permanent health education, intersectoral articulation and institutional support for teams. It also presents possible research pathways for future surveys.

Therefore, this study not only contributes important and novel information for the Nursing practice in maternal death surveillance but also shows the need for an integrated approach that goes beyond individual qualification and incorporates strategies to strengthen management, interprofessional work, organizational culture and working conditions, contributing to reducing maternal mortality and to qualifying women’s health care. Having a strengthened health system with duly trained professionals and which ensures women’s health care in their different life cycles is fundamental to reduce maternal and neonatal mortality, especially in the pregnancy, delivery and peri-natal period *continuum* ^24^ ^),(^ ^38^.

## Conclusion

The variables related to training in maternal death surveillance proved to be essential for Primary Health Care (PHC) nurses’ performance, reinforcing the relevance of permanent education to develop this practice. The study also identified the need to broaden the spaces devoted to discussing death cases in team meetings, as well as to ensure the systematic reinstatement of maternal mortality committee analyses to the PHC level, so as to qualify investigation procedures and improve work processes.

The diverse evidence produced indicates potential to devise adaptations in teams’ work processes and in intersectoral actions, support institutional policies in the permanent education scope and strengthen the assistance provided. Such elements contribute to improving health surveillance quality, aligning with compliance with already established international targets such as 3.1 from the Sustainable Development Goals (SDGs), aimed at reducing the Maternal Mortality Ratio (MMR).

## Data Availability

All data generated or analysed during this study are included in this published article.
