# Supplementary material for: Maternal death surveillance and Primary Health Care nurses’ performance in a Brazilian capital city
Source: Rev Lat Am Enfermagem. 2026 Oct 16;34:e4948. doi: 10.1590/1518-8345.7898.4948 (PMC13674967; doi:10.1590/1518-8345.7898.4948)
Supplement: Translation

# Translation: Vigilância do óbito materno e a atuação de enfermeiros da Atenção Primária à Saúde em uma capital brasileira*

**DOI:** 10.1590/1518-8345.7898.4950

**Authors:** Núria Ananda Parron Giacomelli Pereira, Shaiane de Farias Durval, Albert Schiaveto de Souza, Elen Ferraz Teston, Sonia Silva Marcon, Maria de Lourdes de Souza, Bianca Cristina Ciccone Giacon-Arruda

**Affiliations:** 1 Universidade Federal do Mato Grosso do Sul, Instituto Integrado de Saúde, Campo Grande, MS, Brasil.; 2 Universidade Federal do Mato Grosso do Sul, Instituto de Biociências, Campo Grande, MS, Brasil.; 3 Universidade Estadual de Maringá, Departamento de Enfermagem, Maringá, PR, Brasil.; 4 Universidade Federal de Santa Catarina, Florianópolis, SC, Brasil.

**Keywords:** Mortalidade Materna, Morte Materna, Vigilância em Saúde Pública, Atenção Primária à Saúde, Saúde da Família, Enfermagem

## Abstract

**Objetivo:**

identificar os fatores relacionados à atuação dos enfermeiros da Atenção Primária à Saúde na vigilância do óbito materno.

**Método:**

estudo epidemiológico descritivo-analítico, com 143 enfermeiros de uma capital do centro-oeste brasileiro. Os dados foram coletados por questionário estruturado e autoaplicável, sendo analisados por estatística descritiva e inferencial pelo teste qui-quadrado e pela análise multivariada por meio da regressão logística binária, considerando-se nível de significância de 5%.

**Resultados:**

a maioria dos participantes estava na função atual há menos de cinco anos e não havia participado de capacitação sobre vigilância do óbito materno, mas conhecia o seu objetivo, o comitê municipal de mortalidade materna (e seus objetivos). O tempo na função atual esteve associado à participação em capacitação, à realização de investigação, ao conhecimento sobre o comitê e à participação em suas reuniões. A capacitação esteve associada ao conhecimento sobre o objetivo da investigação, sobre o comitê, seus objetivos e à participação em reuniões. Enfermeiros que participaram de capacitação apresentaram uma razão de chances de 5,82 (IC95%: 2,46-13,75) de já terem realizado uma investigação de óbito materno.

**Conclusão:**

a educação permanente é um fator preditivo para a atuação do enfermeiro e deve ser potencializado por políticas institucionais.

## Destaques

**(1)** A vigilância do óbito materno é uma ação essencial na Atenção Primária em Saúde.

**(2)** O enfermeiro exerce papel crucial na investigação do óbito materno.

**(3)** Capacitar e conhecer o comitê de mortalidade materna influenciam no processo.

**(4)** Discutir os casos de óbito materno nas reuniões de equipe é primordial.

**(5)** É indispensável o retorno das análises do comitê de mortalidade materna à Atenção Primária em Saúde.

## Introdução

A mortalidade materna (MM) é considerada um dos principais desafios da saúde pública mundial. A meta 3.1 dos Objetivos de Desenvolvimento Sustentável (ODS) estabelece como objetivo reduzir a razão de mortalidade materna (RMM) para menos de 70 óbitos por 100 mil nascidos vivos até 2030. Apesar desse compromisso internacional, em 2020, a RMM foi estimada em 223 óbitos por 100 mil nascidos vivos, o que evidencia a necessidade de intensificar esforços para atingir a meta proposta^1^. 

No Brasil, a RMM aumentou de 57,9 em 2019 para 74,7 em 2021, revertendo a tendência de queda observada desde 1990. Esses valores estão distantes da meta nacional, que prevê alcançar uma RMM de 30 até 2030^2^. As principais causas da mortalidade materna (MM) incluem hemorragia obstétrica, transtornos hipertensivos e causas indiretas (condições pré-existentes geralmente agravadas pela gestação). A maioria desses óbitos é evitável e está relacionada a fatores individuais (raça/cor, renda, escolaridade e local de moradia) e contextuais (iniquidades no acesso e na qualidade da assistência à saúde)^3^ ^)-(^ ^4^.

Diante desse contexto, a Organização Mundial da Saúde (OMS) recomenda a implementação da Vigilância e Resposta à Morte Materna e Perinatal (VRMMP) como estratégia para reduzir óbitos evitáveis e compreender os determinantes sociais relacionados à saúde materna^5^ ^)-(^ ^6^. No Brasil, a investigação dos óbitos de mulheres em idade fértil é obrigatória e deve ser realizada por profissionais de saúde, com o apoio dos comitês de mortalidade materna, que reúnem diferentes atores institucionais e da sociedade civil para analisar casos e propor melhorias^7^ ^)-(^ ^8^. 

No entanto, a efetivação da VRMMP ainda enfrenta desafios, sobretudo em países de baixa e média renda, incluindo dificuldades relacionadas à disponibilidade de profissionais capacitados, infraestrutura, financiamento e qualidade dos dados^9^ ^)-(^ ^10^. Estima-se que 34% dos registros de óbitos maternos estejam incompletos e que até 39% das mortes sejam subestimadas devido à subnotificação ou classificação inadequada^11^. No Brasil, a investigação de 88% dos 64 mil óbitos de mulheres em idade fértil registrados em 2019 resultou em um aumento de 32,7% no número de óbitos maternos oficialmente reconhecidos^12^.

No modelo brasileiro de saúde, o enfermeiro da APS é responsável por atividades que abrangem desde a assistência no ciclo gravídico-puerperal (pré-natal, puericultura, avaliação de risco, busca ativa) até a realização de ações educativas e de investigação de óbitos^13^ ^)-(^ ^14^. Nesse cenário, evidencia-se a necessidade da qualificação e aprimoramento da vigilância do óbito materno em seus diferentes aspectos e espaços. A Atenção Primária à Saúde (APS) e seus profissionais, especialmente os enfermeiros, são fundamentais nesse processo^15^.

Frente ao exposto e considerando o papel central do enfermeiro no território e na liderança das equipes de saúde, compreender os fatores que influenciam a participação desse profissional na VRMMP é essencial para qualificar as estratégias de prevenção de óbitos maternos evitáveis, aprimoramento das políticas públicas e dos programas de saúde materna. E, com isso, contribui-se para a redução de um problema que atinge direitos fundamentais das mulheres, como o direito à saúde reprodutiva e sexual, e o acesso a serviços de saúde adequados ao ciclo gravídico-puerperal^14^ ^),(^ ^16^.

Assim, o objetivo deste estudo foi identificar os fatores associados à atuação dos enfermeiros da APS na vigilância do óbito materno. 

## Método

### Tipo de estudo

Estudo epidemiológico descritivo-analítico. Para sua estrutura e organização, seguiram-se as recomendações da declaração *Strengthening the Reporting of Observational Studies in Epidemiology* (STROBE)^17^.

### Local

O estudo foi realizado em unidades de saúde da APS, isto é, a porta de entrada do Sistema Único de Saúde Brasileiro (SUS), vinculadas à secretaria municipal de saúde de uma capital da região centro-oeste do Brasil. No período do estudo, a referida capital contava com 14 Unidades Básicas de Saúde (UBS), 54 Unidades de Saúde da Família (USF) e três Clínicas da Família (CF), totalizando 71 unidades, distribuídas em sete regiões de saúde. Assim, o Comitê Municipal de Prevenção de Mortalidade Materna, Infantil e Fetal (CMPMMIF) foi criado no ano de 2001. 

### Período

O período de coleta de dados foi de dezembro de 2019 a março de 2021, frente às questões organizacionais e epidemiológicas, devido à pandemia da COVID-19. 

### População

A população do estudo foi composta por 204 enfermeiros vinculados às 71 unidades de saúde participantes. A inclusão desses profissionais deve-se ao fato de que, no município investigado, os enfermeiros da APS, conforme preconizado pelos protocolos locais de vigilância, são responsáveis pela condução das visitas e entrevistas domiciliares junto às famílias para a investigação de óbitos de mulheres em idade fértil, como nos casos de óbito materno. 

### Critérios de seleção

Os critérios de inclusão foram: ser enfermeiro, de ambos os sexos, independentemente do tipo de contrato de trabalho (efetivo ou por tempo determinado). Não houve delimitação quanto ao tempo de trabalho ou à experiência relacionada à investigação de óbito materno. Não foram incluídos profissionais que estavam afastados das atividades na unidade de saúde (independentemente do motivo) ou em período de férias no momento da coleta de dados. Por sua vez, foram excluídos os casos de preenchimento incompleto - ausência de resposta em, no mínimo, 10% das questões do instrumento de coleta de dados.

### Definição da amostra

A amostra foi constituída por conveniência, com o intuito de abranger a totalidade do território municipal**,** estratégia relevante para pesquisas sobre processos de trabalho na APS. Entretanto, devido à coincidência da coleta de dados com o período da pandemia de COVID-19, que pode ter impactado a participação dos profissionais, optou-se por realizar o cálculo clássico de tamanho mínimo da amostra para estimar proporções em populações finitas, visando subsidiar a interpretação dos resultados. O resultado indicou um tamanho de amostra mínima de 103 participantes para garantir precisão estatística, considerando 204 enfermeiros elegíveis, nível de confiança de 95% e erro máximo tolerável de 5%^18^. 

Todos os enfermeiros elegíveis foram convidados a participar, seis não foram incluídos por estarem afastados de suas atividades laborais no momento da coleta de dados, e 55 não devolveram o questionário, mesmo após três ou mais tentativas de contato e envio de lembretes pela pesquisadora principal, com ausência de justificativa. A amostra final foi composta por 143 profissionais, número de participantes foi superior ao calculado para amostra mínima, para garantir robustez analítica^18^. Ademais, as taxas de enfermeiros respondentes por cada uma das sete regiões de saúde foram maiores que 60%, conforme indicado na Tabela 1.

**Tabela 1:** Número de enfermeiros lotados na APS* e de participantes da pesquisa, e porcentagem conforme região de saúde. Campo Grande, MS, Brasil, 2019

Região de Saúde	Número de Enfermeiros	Número de Enfermeiros Participantes	%^†^ de Enfermeiros Participantes
Região 1	55	41	74,5
Região 2	36	26	72,2
Região 3	36	23	63,9
Região 4	29	20	69
Região 5	28	18	64,3
Região 6	13	8	61,5
Região 7	7	7	100
Total	204	143	70,1

*APS = Atenção Primária à Saúde; ^†^% = Porcentagem

### Variáveis do estudo

As variáveis utilizadas para descrever a atuação do enfermeiro na vigilância do óbito materno na APS incluíram: realização de investigação do óbito materno enquanto enfermeiro (sim/não); primeira fonte de informação sobre o óbito (familiar/Agente Comunitário de Saúde-ACS/técnico da vigilância em saúde/outros); realização de pré-natal da mulher pelo enfermeiro (sim/não); forma do primeiro contato com a família para a investigação (ACS/contato telefônico/visita domiciliar/outro); presença de outro profissional durante visita domiciliar para investigação (sim/não); recusa da família em participar da investigação (sim/não); suporte fornecido pela equipe de vigilância (sim/não); dificuldades no preenchimento da Ficha de Investigação de Óbito Materno - Serviço de Saúde Ambulatorial (sim/não) e da Ficha de Investigação de Óbito Materno - Entrevista Domiciliar^19^ (sim/não); recebimento das recomendações da análise do comitê de mortalidade materna (sim/não); e discussão do caso em reunião de equipe (sim/não). 

As variáveis relacionadas à capacitação e ao conhecimento dos profissionais incluíram: realização de capacitação em vigilância do óbito materno (sim/não); conhecimento sobre o objetivo da investigação (sim/não); conhecimento sobre a existência do comitê de MM no município (sim/não), sobre seu objetivo (sim/não) e participação em suas reuniões (sim/não); além do tempo de atuação na função atual (em anos completos). 

Para a caracterização sociodemográfica, profissional e de formação, consideraram-se as variáveis: sexo (feminino/masculino); idade (em anos); tipo de unidade de saúde de lotação atual (UBS/UBSF/CF); tempo de formação na graduação em enfermagem (em anos); formação complementar (sim/não/omisso) e respectivo nível (*lato sensu*, *stricto sensu*, ambos ou não informado).

### Instrumentos utilizados para a coleta das informações

A coleta de dados foi realizada por meio de um questionário estruturado e autoaplicável, elaborado pela pesquisadora principal. Para tanto, inicialmente, realizou-se um mapeamento das recomendações do Ministério da Saúde e da literatura sobre vigilância do óbito materno, para definição das dimensões a serem investigadas. A primeira versão do instrumento foi apresentada e discutida com uma pesquisadora com experiência em estudo sobre óbito materno com o objetivo de avaliar a clareza, pertinência e abrangência dos itens, sendo sugeridas pequenas alterações na forma de apresentação de algumas questões e opções de respostas. 

Após isso, solicitou-se a uma enfermeira da APS, com experiência prévia em investigação de óbito materno, que avaliasse e respondesse o questionário, a qual sugeriu apenas alterações nas sequências das questões. A versão final do instrumento foi composta por 34 questões estruturadas, organizadas em três blocos: 1) características sociodemográficas, de trabalho e de formação profissional (oito questões); 2) conhecimento e capacitação sobre a investigação do óbito materno e o comitê de MM (cinco questões); e 3) experiência profissional dos participantes na realização da investigação (21 questões). As variáveis utilizadas para descrever a atuação do enfermeiro na vigilância do óbito materno na APS, neste estudo, integravam esse instrumento e estão descritas no item anterior. 

### Coleta de dados

Para a coleta de dados, a pesquisadora principal solicitou autorização para a divulgação e início da pesquisa às coordenações das sete regiões de saúde do município. Para padronizar a aplicação, a pesquisadora principal entregou pessoalmente um envelope contendo o instrumento de coleta de dados, orientações sobre seu preenchimento e devolução, além de duas vias do Termo de Consentimento Livre e Esclarecido (TCLE). Nesse momento, as mesmas orientações foram fornecidas verbalmente a todos os participantes, de modo a reduzir possíveis vieses de coleta**.**

O instrumento preenchido, juntamente com o TCLE assinado, deveria ser alocado no mesmo envelope, lacrado e entregue à gestão da unidade de saúde, que estava orientada sobre o encaminhamento destes à respectiva coordenação da região de saúde, para posterior recolhimento pela pesquisadora. Entretanto, foram necessários contatos periódicos com as unidades e com os profissionais para reforçar os lembretes sobre a participação no estudo e o preenchimento do instrumento. Dessa forma, o recolhimento dos questionários foi conduzido mensalmente, tanto nas coordenações regionais quanto diretamente nas unidades de saúde. 

Alguns participantes solicitaram um novo envelope contendo o instrumento devido ao extravio do material ou por terem ingressado na equipe após o início da coleta. A referida situação ocorreu, principalmente, devido à realização de concurso público com consequente substituição de profissionais contratados temporariamente por servidores efetivos, bem como à pandemia COVID-19, que demandou a reorganização da assistência e o remanejamento das atividades dos profissionais entre diferentes serviços. 

### Tratamento e análise dos dados

As respostas dos questionários foram duplamente digitadas em uma planilha no Microsoft Excel 2013, para identificação de possíveis inconsistências, e analisadas no *software* estatístico Jamovi^®^, adotando-se nível de significância de 5%. O teste qui-quadrado foi empregado para verificar a associação entre capacitação e conhecimentos dos enfermeiros sobre vigilância do óbito materno. Para identificar fatores associados à realização da investigação de óbito materno (desfecho), realizou-se regressão logística binária, estimando *odds ratios* (OR) ajustadas e intervalos de confiança de 95% (IC95%)^20^.

As variáveis foram preparadas por meio de codificações e categorização, e algumas variáveis foram recodificadas para adequação ao teste utilizado: idade em faixas etárias (18-30, 31-40, 41-50, 51-60 e acima de 61 anos); tempo de formação e tempo de atuação dicotomizados a partir da mediana, com valor de seis anos (menor/maior ou igual a mediana); formação complementar (sim/não) e nível (graduação/pós-graduação); e a forma do primeiro contato com a família para a investigação (presencial - ACS e visita domiciliar/não presencial - telefone e outros). 

Por conseguinte, respeitando-se a ausência de multicolinearidade (examinada pelo *variance inflation factor* e pela tolerância), todas as variáveis independentes foram incluídas no modelo inicial, refinado posteriormente pelo método *backward stepwise* para obtenção do modelo final. A qualidade do ajuste foi avaliada por estatísticas globais (*deviance* e critérios de informação de Akaike e Bayesiano), pseudo-R² (Cox & Snell e Nagelkerke) e teste de razão de verossimilhança^21^. 

Adicionalmente, para avaliar sua robustez e capacidade discriminativa, realizou-se uma análise de desempenho preditivo do modelo final de regressão logística. As métricas de acurácia global, sensibilidade, especificidade, área sob a curva ROC (AUC), e Escore de Brier foram estimadas, o que permitiu examinar a estabilidade do modelo, complementando a avaliação do ajuste global, dada a natureza organizacional e multifatorial do desfecho^19^.

### Aspectos éticos

O estudo foi aprovado pelo Comitê de Ética em Pesquisa com seres humanos da instituição signatária, conforme CAAE 22855519.1.0000.0021.

## Resultados

Dos 143 enfermeiros participantes do estudo, a maioria era do sexo feminino (n=130; 90,9%) e com idade entre 18 e 60 anos e com tempo na função menor que seis anos completos (n=101; 71,6%). Em relação à unidade de lotação, 74,8% estavam lotados em USF (n=107), 17,5% em UBS (n=25) e 7,7% em CF (n=11). Quanto à formação, 65,8% (n=94) possuíam até 10 anos de graduação em enfermagem e 79,6% (n=110) apresentavam formação complementar em nível de pós-graduação, predominantemente na modalidade *lato sensu* (n=103; 72%).

Sobre a investigação do óbito materno, 58,7% (n=84) relataram já ter realizado ao menos uma investigação, não sendo observadas diferenças estatisticamente significativas quanto ao tipo de unidade onde atua e à participação em capacitação (p=0,396), realização da investigação de óbito materno (p = 0,942) nem quanto à discussão de casos em equipe (p = 0,297). A principal fonte de informação sobre o óbito foi o ACS (n=34; 41,5%), seguida por técnicos da vigilância em saúde (n=24; 29,3%) e por familiares (n=18; 22%). 

A maioria dos enfermeiros não eram responsáveis pelo pré-natal da gestante falecida (n=59; 70,2%). A forma mais comum de primeiro contato com a família para a investigação foi a visita domiciliar (n=42; 52,5%), frequentemente acompanhada de outro profissional da equipe (n=62; 76,5%). Quanto ao suporte institucional, 53,6% (n=45) relataram apoio da equipe de vigilância, enquanto 46,4% (n=39) indicaram ausência de suporte.

A dificuldade no preenchimento das fichas de investigação foi apontada por 11% dos participantes e apenas um enfermeiro (1,2%) informou ter recebido recomendações do comitê de MM após a análise do óbito. A discussão dos casos em reuniões de equipe foi referida por 32,9% (n=27) dos profissionais, e 30,5% (n=25) relataram a recusa da família em participar da investigação domiciliar.

Embora 34,3% (n=49) dos enfermeiros tenham participado de capacitação específica sobre vigilância do óbito materno, a maioria relatou conhecer o objetivo da investigação (n=121; 84,6%), bem como a existência do comitê de mortalidade materna no município (n=100; 65,9%) e o seu objetivo (n=100; 69,9%). No entanto, apenas 17,5% (n=25) participaram das reuniões do comitê.

A variável tempo na função apresentou associação significativa com a participação em capacitação sobre vigilância do óbito materno (p<0,001), com o conhecimento sobre a existência do comitê de mortalidade materna (p<0,001) e com a participação em reunião do comitê de mortalidade materna (p=0,005) (Tabela 2). 

**Tabela 2:** Associação entre variáveis do estudo e o tempo na função atual (n* = 143). Campo Grande, MS, Brasil, 2019-2021

Variável	Tempo na função atual (APS^†^)		
	< 6 anos	6 anos ou mais	Valor de p^‡^
	n* (%)^§^	n* (%)^§^	
Capacitação em vigilância do óbito materno			
Sim	21 (20,8)	28 (70)	<0,001
Não	80 (79,2)	12 (30)	
Conhecimento sobre o comitê de mortalidade materna no município			
Sim	62 (62,6)	37 (92,5)	<0,001
Não	37 (37,4)	3 (7,5)	
Conhecimento do objetivo do comitê de mortalidade materna no município			
Sim	64 (66)	35 (89,7)	0,005
Não	9 (29,0)	24 (36,4)	
Participação em reunião do comitê			
Sim	4 (12,1)	13 (32,5)	0,004
Não	89 (88,1)	27 (67,5)	
Conhecimento sobre o objetivo da investigação do óbito materno			
Sim	84 (85,7)	36 (92,3)	0,291
Não	14 (14,3)	3 (7,7)	

*n = Tamanho da amostra composta pelos enfermeiros; ^†^APS = Atenção Primária à Saúde; ^‡^Teste Qui-quadrado, considerando um nível de significância de 5%; ^§^% = Porcentagem

A participação em capacitação sobre vigilância do óbito materno apresentou associações significativas com as variáveis relacionadas ao conhecimento sobre o objetivo da investigação do óbito (p=0,025), à existência do comitê de mortalidade materna (p<0,001) e ao seu objetivo (p<0,001), bem como à participação em alguma reunião do comitê (p=0,003), conforme demonstrado na Tabela 3.

**Tabela 3:** Associação entre variáveis do estudo e a capacitação em vigilância do óbito materno (n* = 143). Campo Grande, MS, Brasil, 2019-2021

Variável	Capacitação em vigilância do óbito materno		
	Sim	Não	Valor de p^‡^
	n*(%^†^)	n*(%^†^)	
Conhecimento sobre o comitê de mortalidade materna no município			
Sim	46 (93,9)	54 (58,7)	<0,001
Não	3 (6,1)	38 (41,3)	
Conhecimento do objetivo do comitê de mortalidade materna no município			
Sim	45 (93,8)	55 (61,1)	<0,001
Não	3 (6,3)	35 (38,9)	
Participação em reunião do comitê			
Sim	15 (30,6)	10 (10,6)	0,003
Não	34 (69,4)	84 (89,4)	
Conhecimento sobre o objetivo da investigação do óbito materno			
Sim	46 (95,8)	75 (82,4)	0,025
Não	2 (4,2)	16 (17,6)	
Dificuldade no preenchimento Ficha de Investigação de Óbito Materno - M1^§^			
Sim	2 (5,0)	7 (16,7)	0,091
Não	38 (95,0)	35 (83,3)	
Dificuldade no preenchimento Ficha de Investigação de Óbito Materno - M3^||^			
Sim	3 (7,5)	6 (14,3)	0,326
Não	37 (92,5)	36 (85,7)	

*n = Tamanho da amostra composta pelos enfermeiros; ^†^% = Porcentagem; ^‡^Teste Qui-quadrado, considerando um nível de significância de 5%; ^§^M1 = Ficha de Investigação de Óbito Materno - Serviço de Saúde Ambulatorial; ^||^M3 = Ficha de Investigação de Óbito Materno - Entrevista Domiciliar

Por sua vez, o conhecimento sobre a existência do comitê de mortalidade materna esteve significativamente associado ao conhecimento sobre o objetivo do comitê, à participação em reuniões e ao maior conhecimento sobre o objetivo da investigação do óbito materno (Tabela 4). 

**Tabela 4:** Associação entre variáveis do estudo e o conhecimento sobre a existência do comitê de mortalidade materna no município (n* = 143). Campo Grande, MS, Brasil, 2019-2021

Variável	Conhecimento sobre o comitê de mortalidade materna no município		
	Sim	Não	Valor de p^‡^
	n*(%^†^)	n*(%^†^)	
Conhecimento do objetivo do comitê de mortalidade materna no município			
Sim	84 (87,5)	14 (35,0)	<0,001
Não	12 (12,5)	26 (65,0)	
Participação em reunião do comitê			
Sim	24 (24,0)	1 (2,4)	0,002
Não	76 (76,0)	40 (97,6)	
Conhecimento sobre o objetivo da investigação do óbito materno			
Sim	90 (92,8)	29 (72,5)	0,001
Não	7 (7,2)	11 (27,5)	

*n = Tamanho da amostra composta pelos enfermeiros; ^†^% = Porcentagem; ^‡^Teste Qui-quadrado, considerando um nível de significância de 5%

A Tabela 5 apresenta os coeficientes do modelo final de regressão logística binária. Observa-se que o intercepto não foi estatisticamente significativo (β = -0,128; SE = 0,207; Z = -0,618; p = 0,536). Em contraste, a participação em capacitação mostrou-se fortemente associada à realização de investigação de óbito materno, com coeficiente positivo significativo (β = 1,762; SE = 0,438; Z = 4,020; p < 0,001). Essa estimativa corresponde a uma razão de chances de 5,82 (IC 95%: 2,46-13,75), indicando que profissionais que participaram de capacitação apresentaram probabilidade quase seis vezes maior de já terem realizado uma investigação, quando comparados aos não capacitados.

**Tabela 5:** Coeficientes do modelo final de regressão logística binária, entre variáveis profissionais e operacionais e a realização prévia de investigação de óbito materno por enfermeiros da Atenção Primária à Saúde. Campo Grande, MS, Brasil, 2019-2021

Preditor	β^*^	SE^†^	Z^‡^	p^§^	OR^||^ (IC 95%^¶^)
Intercepto	-0,128	0,207	-0,618	0,536	0,88 (0,58 - 1,32)
Capacitação em vigilância do óbito materno					
Sim - Não	1,762	0,438	4,020	<0,001	5,82 (2,46 - 13,75)
Desviância = 174; AIC^**^ = 178; R²CS^††^ = 0,128; R²N^‡‡^ = 0,173; VIF^§§^ = 1,00; tolerância = 1,00					

*β = Coeficiente estimado no modelo logístico (logito); ^†^SE = Erro-padrão da estimativa; ^‡^Z = Estatística do teste de Wald; ^§^p = Nível de significância; ^||^OR= Razão de chances (*odds ratio*); ^¶^IC 95% = Intervalo de confiança de 95%; **AIC = Critério de Informação de Akaike; ^††^R²CS = Coeficiente de determinação de Cox & Snell; ^‡‡^R²N = Coeficiente de Determinação de Nagelkerke; ^§§^VIF = *Variance Inflation Factor* (fator de inflação da variância)

O modelo de regressão logística apresentou ajuste global satisfatório, evidenciado pelo valor da desviância igual a 174 e pelo Critério de Informação de Akaike (AIC = 178), sugerindo boa parcimônia entre complexidade e capacidade preditiva. As medidas de pseudo-R² indicaram desempenho moderado do modelo, com R² de Cox & Snell igual a 0,128 e R² de Nagelkerke corrigido para 0,173. A avaliação dos pressupostos do modelo não evidenciou colinearidade, uma vez que o Fator de Inflação da Variância (VIF = 1,00) e a tolerância (1,00) indicaram total independência entre o preditor incluído e ausência de redundância estrutural que pudesse comprometer a estabilidade das estimativas.

A análise de desempenho preditivo indicou acurácia de 63,6%, com área sob a curva ROC (AUC) de 0,672, sugerindo capacidade discriminativa moderada. O modelo apresentou elevada especificidade (86,2%) e sensibilidade moderada (48,2%), indicando maior capacidade para identificar corretamente profissionais que não haviam realizado a investigação de óbito materno. O Escore Brier foi de 0,210, evidenciando erro de calibração aceitável e estabilidade das probabilidades estimadas.

## Discussão

Os resultados deste estudo indicaram que a atuação do enfermeiro da APS na vigilância do óbito materno está diretamente relacionada à participação em capacitação específica sobre o tema, configurando-se como o único fator significativamente relacionado à realização prévia de investigações de óbito materno. A magnitude dessa associação, com probabilidade quase seis vezes maior entre profissionais capacitados, reforça a centralidade na educação permanente como elemento estruturante para o fortalecimento da vigilância em saúde e para a qualificação das práticas investigativas no âmbito da APS^22^.

Apesar de a maioria dos enfermeiros participantes possuir formação complementar em nível de pós-graduação *lato sensu*, observou-se uma baixa proporção de profissionais que participaram de capacitações específicas sobre vigilância do óbito materno. Ainda, a ausência de diferenças entre os tipos de unidade de atuação indica que as fragilidades relacionadas à discussão de casos e à investigação do óbito materno não se restringem a um modelo específico de serviço, mas refletem desafios organizacionais transversais à atenção primária. 

Esse achado sugere que a formação acadêmica generalista, ainda que avançada, não é suficiente para garantir o desenvolvimento das competências técnicas, analíticas e operacionais necessárias à condução qualificada da investigação de óbitos, corroborando evidências de que a vigilância da mortalidade materna demanda conhecimentos específicos, atualizados e alinhados às diretrizes nacionais e internacionais^23^.

A educação permanente, nesse contexto, contribui para o aprimoramento da autonomia técnica e da segurança profissional, favorecendo o acesso às diretrizes nacionais e internacionais e a incorporação de práticas alinhadas às políticas públicas voltadas à redução da mortalidade materna. Embora a ausência de capacitação não indique desconhecimento dos princípios da vigilância do óbito materno, os resultados sugerem que a expertise necessária para conduzir investigações não se constrói exclusivamente com o tempo de prática ou com a formação acadêmica, mas exige inserção em processos formativos específicos, orientados à vigilância e à qualificação da atenção^22^ ^)-(^ ^23^. 

A participação em capacitação mostrou-se associada ao maior tempo de atuação na APS, ao conhecimento sobre a existência e os objetivos do comitê de mortalidade e à participação em reuniões desse colegiado. Esses resultados indicam que o tempo de atuação pressupõe experiência acumulada, o que pode ampliar a exposição dos profissionais a processos formativos e organizacionais relacionados à vigilância e favorecer o desenvolvimento de competências específicas. No entanto, os achados demonstram que o tempo de serviço, isoladamente, não garante a efetiva participação na vigilância do óbito materno, reforçando a necessidade de estratégias institucionais sistemáticas de educação permanente.

A capacitação específica possibilita aproximação com as etapas essenciais do processo de vigilância do óbito, potencializando a coleta e a obtenção de informações importantes para a análise crítica do caso pelas demais instâncias da rede, como o comitê de MM e a equipe de vigilância em saúde^24^. A qualidade dos dados registrados em cada ponto da rede de assistência e disponibilizados pelos sistemas de informação em saúde está diretamente relacionada a subnotificação e ao sub-registro^25^, o que compromete a elaboração de recomendações e o potencial preventivo da vigilância. 

O modelo de regressão logística apresentou desempenho explicativo moderado, conforme indicado pelos valores de R² de Cox & Snell e de Nagelkerke, o que reflete a natureza multifatorial e organizacional da vigilância do óbito materno, e evidencia que, embora a capacitação profissional se configure como fator central, existem outros determinantes relevantes não contemplados nas variáveis analisadas. 

Fatores institucionais, organizacionais e contextuais como a disponibilidade de recursos humanos, o apoio institucional, a rotatividade de profissionais, a carga de trabalho, a cultura organizacional e as condições de saúde mental dos trabalhadores podem exercer influência significativa sobre a realização das investigações e devem ser considerados em estudos futuros. Desta forma, embora a capacitação tenha se destacado como fator central, ela não deve ser compreendida de forma isolada, mas integrada a um conjunto mais amplo de intervenções sistêmicas^23^.

Entre os achados operacionais, destaca-se o papel do ACS como primeira fonte de informação sobre o óbito materno, bem como o uso da visita domiciliar como estratégia predominante de primeiro contato com a família, frequentemente realizada com a participação de mais de um profissional da equipe. Essas práticas são alinhadas aos princípios da APS e da Vigilância em Saúde, especialmente no que se refere ao vínculo prévio com a família da gestante à identificação precoce de eventos e à abordagem interprofissional^18^ ^),(^ ^26^. O ACS, por sua inserção comunitária e vínculo prévio com as famílias, desempenha papel estratégico na detecção e notificação de óbitos, reforçando a importância de sua capacitação e articulação com os enfermeiros, que atuam como coordenadores das equipes^27^.

A APS pode ser operacionalizada por diferentes modelos de atenção e arranjos organizacionais desde que preservem atributos essenciais da APS como o primeiro contato e a longitudinalidade, integralidade e coordenação do cuidado^25^. No município estudado, apesar da APS ser composta por unidades de saúde de diferentes tipos de unidades (USF, UBS e CF), todas são responsáveis pela investigação do óbito materno no território adscrito, o que corrobora com o resultado deste estudo que não indicou diferenças estatisticamente significativas quanto ao tipo de unidade de atuação e a participação em capacitação, realização da investigação de óbito materno e discussão de casos em equipe.

Apesar dessas potencialidades, o estudo revelou fragilidades operacionais críticas no processo de vigilância do óbito materno. O dado de que apenas um enfermeiro recebeu recomendações do comitê de mortalidade materna após a análise do óbito é particularmente preocupante, pois compromete o caráter pedagógico, preventivo e transformador da vigilância. A ausência de devolutiva sistemática fragiliza a retroalimentação dos serviços, limita a incorporação de aprendizados institucionais e reduz o potencial das recomendações para a reorganização dos processos de trabalho e a melhoria da qualidade da assistência^24^ ^)-(^ ^25^.

De forma semelhante, a baixa proporção de profissionais que relataram discutir os casos de óbito materno em reuniões de equipe evidencia a fragilidade dos espaços coletivos de reflexão e análise crítica do cuidado. A ausência dessas discussões dificulta a identificação de falhas assistenciais evitáveis, compromete a tomada de decisões compartilhadas e enfraquece o aprendizado organizacional^28^. 

No âmbito internacional, fatores como falta de tempo, liderança e suporte institucional, comunicação limitada, papéis profissionais pouco claros e recursos restritos também tendem a dificultar a colaboração interprofissional em serviços de saúde^29^ ^)-(^ ^32^. Esse cenário pode estar relacionado à sobrecarga de trabalho, à falta de tempo protegido para reuniões, à insuficiente valorização dessas práticas no cotidiano da APS e à fragilidade de uma cultura organizacional voltada à análise de eventos adversos^33^. 

Ademais, a sobrecarga assistencial e o desgaste físico e emocional dos profissionais, intensificados no contexto pandêmico, também devem ser considerados como fatores que podem ter influenciado negativamente o engajamento em atividades de vigilância, educação permanente e discussão em equipe. O *burnout* e o sofrimento psíquico dos trabalhadores de saúde têm sido amplamente descritos na literatura como elementos que afetam a qualidade do cuidado e a adesão a práticas institucionais, devendo ser incorporados também às análises sobre a vigilância do óbito materno^25^ ^),(^ ^34^ ^)-(^ ^35^.

Outro achado relevante refere-se ao suporte institucional, uma vez que quase metade dos enfermeiros em estudo relatou não receber apoio da equipe de vigilância em saúde. Essa lacuna evidencia fragilidades na articulação entre a APS e os demais componentes do sistema de vigilância, podendo gerar insegurança profissional, dificuldades operacionais e descontinuidade das ações investigativas. O suporte técnico e institucional é fundamental para garantir a qualidade das investigações, especialmente em contextos de maior vulnerabilidade social e complexidade assistencial^25^.

A ausência de acompanhamento pré-natal por parte de parcela significativa dos enfermeiros investigantes também merece destaque. O pré-natal é um momento fundamental para a identificação precoce de riscos obstétricos, realização de intervenções oportunas e fortalecimento do vínculo com a gestante. Falhas nesse acompanhamento podem refletir problemas organizacionais, limitações de acesso, insuficiência de recursos, bem como fatores sociais e culturais que impactam a adesão das mulheres aos serviços^36^ ^)-(^ ^37^. A resistência ou recusa da família em participar da investigação domiciliar pode estar relacionada à percepção da qualidade da assistência recebida e à trajetória da mulher na rede de atenção.

Contudo, esses resultados devem ser interpretados considerando o início do estado pandêmico pela COVID-19, no contexto brasileiro. Esse evento, não controlável no planejamento do estudo, transpassou quase que a totalidade do processo de coleta de dados e provocou transformações no funcionamento da APS. A reorganização dos serviços, a priorização de ações emergenciais, a suspensão ou redução de atividades coletivas assistenciais e organizacionais, a limitação de visitas domiciliares, entre outras alterações, pode ter contribuído para a baixa adesão às etapas do processo de vigilância observadas no estudo, principalmente nos processos de educação permanente, de discussão em equipe e no retorno institucional das análises dos comitês. 

O contexto pandêmico também deve ser considerado em relação as limitações desse estudo. A taxa de não resposta de aproximadamente 30% dos profissionais elegíveis, possivelmente relacionada à sobrecarga assistencial nesse período, associada a amostra por conveniência e ao desconhecimento de informações sobre os não respondentes, pode introduzir viés de resposta, uma vez que profissionais mais sobrecarregados ou menos envolvidos com a vigilância podem ter sido menos propensos a participar, possivelmente superestimando o nível de conhecimento e engajamento observado.

Estas limitações somadas à realização do estudo em um único município e ao desempenho explicativo moderado do modelo, indicam a necessidade de investigações futuras que incorporem variáveis organizacionais, institucionais e psicossociais, bem como análises estratificadas por tipo de unidade de saúde, considerando as especificidades dos diferentes modelos de organização da APS. 

Apesar dessas limitações, o estudo conseguiu abranger profissionais de todas as regiões de saúde do município e teve um quantitativo de participantes superior à amostra mínima necessária para garantir a robustez analítica. Os achados, mesmo que não representativos devido os vieses de seleção da amostra, evidenciam, de maneira consistente^18^, fatores modificáveis que podem subsidiar gestores, de diferentes contextos, na formulação de políticas institucionais voltadas ao fortalecimento da educação permanente em saúde, da articulação intersetorial e do suporte institucional às equipes. E apresenta possíveis caminhos investigativos para pesquisas futuras. 

Portanto, este estudo traz informações importantes e inéditas não apenas para a prática de enfermagem na vigilância do óbito materno, mas demonstra que é necessária uma abordagem integrada, que ultrapasse a qualificação individual e incorpore estratégias de fortalecimento da gestão, do trabalho interprofissional, da cultura organizacional e das condições de trabalho, contribuindo para a redução da mortalidade materna e para a qualificação da atenção à saúde da mulher. Um sistema de saúde fortalecido, com profissionais capacitados e que garanta assistência à saúde da mulher em seus diferentes ciclos de vida, sobretudo no *continuum* da gravidez, parto, pós-parto e período perinatal é fundamental para a diminuição da MM e neonatal^24^ ^),(^ ^38^. 

## Conclusão

As variáveis relacionadas à capacitação em vigilância do óbito materno mostraram-se essenciais para a atuação dos enfermeiros na Atenção Primária à Saúde (APS), reforçando a relevância da educação permanente para o desenvolvimento dessa prática. O estudo também identificou a necessidade de ampliar os espaços de discussão dos casos de óbito entre as equipes, bem como de assegurar o retorno sistemático das análises dos comitês de mortalidade materna à APS, de modo a qualificar as investigações e aprimorar o processo de trabalho.

As evidências produzidas apontam potencial para orientar ajustes nos processos de trabalho das equipes e nas ações intersetoriais, subsidiar políticas institucionais de educação permanente e fortalecer a assistência. Tais elementos contribuem para o aprimoramento e a qualidade da vigilância em saúde, alinhando-se ao cumprimento de metas internacionais estabelecidas, como a meta 3.1 dos Objetivos de Desenvolvimento Sustentável (ODS) para redução da razão de mortalidade materna (RMM).

## Data Availability

Todos os dados gerados ou analisados durante este estudo estão incluídos neste artigo publicado.

# Translation: Vigilancia de muertes maternas y papel de los enfermeros de la Atención Primaria de Salud en una capital brasileña*

**DOI:** 10.1590/1518-8345.7898.4947

**Authors:** Núria Ananda Parron Giacomelli Pereira, Shaiane de Farias Durval, Albert Schiaveto de Souza, Elen Ferraz Teston, Sonia Silva Marcon, Maria de Lourdes de Souza, Bianca Cristina Ciccone Giacon-Arruda

**Affiliations:** 1 Universidade Federal do Mato Grosso do Sul, Instituto Integrado de Saúde, Campo Grande, MS, Brasil.; 2 Universidade Federal do Mato Grosso do Sul, Instituto de Biociências, Campo Grande, MS, Brasil.; 3 Universidade Estadual de Maringá, Departamento de Enfermagem, Maringá, PR, Brasil.; 4 Universidade Federal de Santa Catarina, Florianópolis, SC, Brasil.

**Keywords:** Mortalidad Materna, Muerte Materna, Vigilancia en Salud Pública, Atención Primaria de Salud, Salud de la Familia, Enfermería

## Abstract

**Objetivo:**

identificar los factores relacionados con el desempeño de los enfermeros de Atención Primaria de Salud la vigilancia de la mortalidad materna.

**Método:**

estudio epidemiológico descriptivo-analítico con 143 enfermeros de una capital de la región Centro-Oeste de Brasil. Los datos se recolectaron mediante un cuestionario estructurado y autoadministrado y se analizaron mediante estadística descriptiva e inferencial mediante prueba de chi-cuadrado y análisis multivariado por regresión logística binaria, considerando un nivel de significancia del 5%.

**Resultados:**

la mayoría de los participantes llevaban menos de cinco años en su puesto actual y no habían participado en capacitación sobre vigilancia de la mortalidad materna, pero estaban familiarizados con su propósito, el comité municipal de mortalidad materna (y sus objetivos). El tiempo en el puesto actual se asoció con la participación en la capacitación, la realización de investigaciones, el conocimiento sobre el comité y la participación en sus reuniones. La capacitación se asoció con el conocimiento sobre el propósito de la investigación, el comité, sus objetivos y la participación en las reuniones. Los enfermeros que participaron en la capacitación tuvieron una razón de probabilidades de 5,82 (IC 95%: 2,46-13,75) de haber realizado previamente una investigación sobre muerte materna.

**Conclusión:**

la formación continua es un factor predictivo del desempeño de los enfermeros y debería potenciarse mediante políticas institucionales.

## Destacados

**(1)** La vigilancia de las muertes maternas es una acción esencial en la Atención Primaria de Salud.

**(2)** Los enfermeros desempeñan un papel crucial en la investigación de la muerte materna.

**(3)** La capacitación y la comprensión del comité de mortalidad materna influyen en el proceso.

**(4)** Es fundamental abordar los casos de muerte materna en las reuniones de equipo.

**(5)** Es fundamental que los análisis del comité de mortalidad materna vuelvan a la Atención Primaria de Salud.

## Introducción

La mortalidad materna (MM) se considera uno de los principales desafíos para la salud pública mundial. La meta 3.1 de los Objetivos de Desarrollo Sostenible (ODS) establece el objetivo de reducir la tasa de mortalidad materna (TMM) a menos de 70 muertes por cada 100 000 nacidos vivos para 2030. A pesar de este compromiso internacional, en 2020, la TMM se estimó en 223 muertes por cada 100 000 nacidos vivos, lo que subraya la necesidad de intensificar los esfuerzos para alcanzar la meta propuesta^1^.

En Brasil, la TMM aumentó de 57,9 en 2019 a 74,7 en 2021, y revirtió la tendencia descendente observada desde 1990. Estos valores están lejos de la meta nacional, que busca alcanzar una TMM de 30 para 2030^2^. Las principales causas de mortalidad materna (MM) incluyen hemorragia obstétrica, trastornos hipertensivos y causas indirectas (condiciones preexistentes generalmente agravadas por el embarazo). La mayoría de estas muertes son evitables y se relacionan con factores individuales (raza/etnia, ingresos, educación y lugar de residencia) y factores contextuales (desigualdades en el acceso y la calidad de la atención de salud)^3^ ^)-(^ ^4^.

Dado este contexto, la Organización Mundial de la Salud (OMS) recomienda la implementación de la Vigilancia y Respuesta a la Muerte Materna y Perinatal (VRMMP) como una estrategia para reducir las muertes evitables y comprender los determinantes sociales relacionados con la salud materna^5^ ^)-(^ ^6^. En Brasil, la investigación de las muertes de mujeres en edad fértil es obligatoria y debe ser llevada a cabo por profesionales de la salud, con el apoyo de los comités de mortalidad materna, que reúnen a diferentes actores institucionales y a la sociedad civil para analizar los casos y proponer mejoras^7^ ^)-(^ ^8^.

Sin embargo, la implementación de la VRMMP aún enfrenta desafíos, especialmente en países de ingresos bajos y medios, incluyendo dificultades relacionadas con la disponibilidad de profesionales capacitados, infraestructura, financiamiento y calidad de datos^9^ ^)-(^ ^10^. Se estima que el 34% de los registros de muertes maternas están incompletos y que hasta el 39% de las muertes se subestiman debido a la subnotificación o clasificación inadecuada^11^. En Brasil, la investigación del 88% de las 64 000 muertes de mujeres en edad fértil registradas en 2019 resultó en un aumento del 32,7% en el número de muertes maternas reconocidas oficialmente^12^.

En el modelo de salud brasileño de salud, el enfermero de atención primaria es responsable de actividades que abarcan desde la atención durante el ciclo embarazo-puerperio (atención prenatal, cuidado infantil, evaluación de riesgos, detección activa de casos) hasta la realización de acciones educativas y la investigación de muertes^13^ ^)-(^ ^14^. En este contexto, se evidencia la necesidad de cualificación y mejora de la vigilancia de la mortalidad materna en sus diferentes aspectos y ámbitos. La Atención Primaria de Salud (APS) y sus profesionales, especialmente los enfermeros, son fundamentales en este proceso^15^.

En vista de lo anterior y considerando el papel central de los enfermeros en la comunidad y en la dirección de los equipos de salud, es esencial comprender los factores que influyen en la participación de este profesional en la VRMMP para mejorar las estrategias de prevención de muertes maternas evitables, así como para fortalecer las políticas públicas y los programas de salud materna. Esto contribuye a reducir un problema que afecta los derechos fundamentales de las mujeres, como el derecho a la salud reproductiva y sexual, y el acceso a servicios de salud adecuados al ciclo gravídico-puerperal^14^ ^),(^ ^16^.

Por lo tanto, el objetivo de este estudio fue identificar los factores asociados con el desempeño de los enfermeros de la Atención Primaria de Salud en la vigilancia de la mortalidad materna.

## Método

### Tipo de estudio

Estudio epidemiológico descriptivo-analítico. Su estructura y organización siguieron las recomendaciones de *Strengthening the Reporting of Observational Studies in Epidemiology* (STROBE)^17^.

### Escenario del estudio

El estudio se realizó en unidades de salud de la APS, que opera como puerta de acceso al Sistema Único de Salud (SUS) brasileño, vinculadas al departamento de salud municipal de una capital de la región Centro-Oeste de Brasil. Durante el período de estudio, dicha capital contaba con 14 Unidades Básicas de Salud (UBS), 54 Unidades de Salud de la Familia (USF) y 3 Clínicas de la Familia (CF), con un total de 71 unidades, distribuidas en siete regiones sanitarias. En ese marco, en 2001 se creó el Comité Municipal para la Prevención de la Mortalidad Materna, Infantil y Fetal (CMPMMIF).

### Período

El período de recopilación de datos abarcó desde diciembre de 2019 hasta marzo de 2021, teniendo en cuenta los problemas organizativos y epidemiológicos derivados de la pandemia de COVID-19.

### Población

La población de estudio estuvo compuesta por 204 enfermeros vinculados a las 71 unidades de salud participantes. Estos profesionales fueron incluidos porque, en el municipio investigado, los enfermeros de la APS, según lo recomendado por los protocolos de vigilancia locales, son los encargados de las visitas domiciliarias y entrevistas con familiares para investigar las muertes de mujeres en edad fértil, como ocurre en los casos de muerte materna.

### Criterios de selección

Los criterios de inclusión fueron: ser profesional de enfermería, sin distinción de sexo, independientemente del tipo de contrato laboral (por tiempo indefinido o temporal). No se estableció ninguna limitación en cuanto a la antigüedad o la experiencia relacionada con la investigación de la muerte materna. No se incluyeron los profesionales que estuviesen apartados de la unidad de salud (sea cual fuere el motivo) o de vacaciones en el momento de la recolección de datos. Asimismo, se excluyeron los casos de formularios incompletos, es decir, aquellos en los que faltaba respuesta en al menos el 10% de las preguntas del cuestionario.

### Definición de la muestra

La muestra se constituyó por conveniencia, con el objetivo de cubrir todo el territorio municipal**,** estrategia relevante para la investigación sobre procesos de trabajo en la APS. Sin embargo, dada la coincidencia de la recolección de datos con el período de la pandemia de COVID-19, que pudo haber impactado la participación de los profesionales, se decidió realizar el cálculo clásico del tamaño mínimo de la muestra para estimar proporciones en poblaciones finitas, con el fin de respaldar la interpretación de los resultados. El resultado indicó un tamaño mínimo de muestra de 103 participantes para garantizar la precisión estadística, considerando 204 enfermeros elegibles, un nivel de confianza del 95% y un error máximo tolerable del 5%^18^.

Todos los enfermeros elegibles fueron invitados a participar; 6 fueron excluidos por hallarse ausentes de sus actividades laborales al momento de la recolección de datos, y 55 no devolvieron el cuestionario, incluso después de tres o más intentos de contacto y recordatorios por parte del investigador principal, sin justificación alguna. La muestra final estuvo compuesta por 143 profesionales; el número de participantes fue superior al indicado como tamaño mínimo de muestra para garantizar la solidez analítica^18^. Además, las tasas de respuesta de los enfermeros para cada una de las siete regiones sanitarias superaron el 60%, como se muestra en la Tabla 1.

**Tabla 1:** Cantidad de enfermeros que prestan servicios en APS* y de participantes en la investigación, y porcentaje según la región sanitaria. Campo Grande, MS, Brasil, 2019

Región Sanitaria	Cantidad de Enfermeros	Cantidad de Enfermeros Participantes	%^†^ de Enfermeros Participantes
Región 1	55	41	74,5
Región 2	36	26	72,2
Región 3	36	23	63,9
Región 4	29	20	69
Región 5	28	18	64,3
Región 6	13	8	61,5
Región 7	7	7	100
Total	204	143	70,1

*APS = Atención Primaria de Salud; ^†^% = Porcentaje

### Variables del estudio

Las variables utilizadas para describir el rol del enfermero en la vigilancia de la muerte materna en la APS incluyeron: realización de investigación de muerte materna como enfermero (sí/no); primera fuente de información sobre la muerte (familiar/Agente Comunitario de Salud - ACS/técnico de vigilancia en salud/otro); atención prenatal proporcionada a la mujer por el enfermero (sí/no); método de primer contacto con la familia para la investigación (ACS/contacto telefónico/visita domiciliaria/otro); presencia de otro profesional durante la visita domiciliaria para la investigación (sí/no); negativa de la familia a participar en la investigación (sí/no); soporte brindado por el equipo de vigilancia (sí/no); dificultades para completar el Formulario de Investigación de Muerte Materna - Servicio de Salud Ambulatorio (sí/no) y el Formulario de Investigación de Muerte Materna - Entrevista Domiciliaria^19^ (sí/no); recepción de recomendaciones del análisis del comité de mortalidad materna (sí/no); y discusión del caso en una reunión de equipo (sí/no).

Las variables relacionadas con la formación y el conocimiento de los profesionales incluyeron: capacitación en vigilancia de la mortalidad materna (sí/no); conocimiento sobre el objetivo de la investigación (sí/no); conocimiento sobre la existencia del comité de mortalidad materna en el municipio (sí/no), sobre su objetivo (sí/no) y participación en sus reuniones (sí/no); además del tiempo de permanencia en el puesto actual (en años completos).

Para la caracterización sociodemográfica, profesional y educativa, se consideraron las siguientes variables: sexo (femenino/masculino); edad (en años); tipo de unidad de salud actual (UBS/UBSF/CF); tiempo transcurrido desde la graduación en enfermería (en años); formación complementaria (sí/no/omitida) y nivel respectivo (*lato sensu*, *stricto sensu*, ambos o no informado).

### Instrumentos utilizados para la recopilación de datos

La recopilación de datos se realizó mediante un cuestionario estructurado y autoadministrado, desarrollado por la investigadora principal. En primer lugar, se revisaron las recomendaciones del Ministerio de Salud y la bibliografía sobre vigilancia de la mortalidad materna para definir las dimensiones a investigar. La primera versión del instrumento se presentó y debatió con una investigadora con experiencia en estudios sobre mortalidad materna, con el objetivo de evaluar la claridad, la pertinencia y la exhaustividad de los ítems, y sugirieron pequeños cambios en la forma de presentar algunas preguntas y opciones de respuesta.

Luego, se solicitó a una enfermera de APS con experiencia previa en investigaciones de muertes maternas que evaluara y respondiera el cuestionario, quien apenas sugirió cambios en la secuencia de las preguntas. La versión final del instrumento consideró 34 preguntas estructuradas, organizadas en tres secciones: 1) características sociodemográficas, laborales y de formación profesional (ocho preguntas); 2) conocimientos y formación sobre investigaciones de muertes maternas y el comité de muertes maternas (cinco preguntas); y 3) experiencia profesional de los participantes en la realización de investigaciones (21 preguntas). Las variables utilizadas para describir el rol del enfermero en la vigilancia de muertes maternas en APS en este estudio se incluyeron en este instrumento y se hallan descriptas en el ítem anterior.

### Recopilación de datos

Para la recopilación de datos, la investigadora principal solicitó autorización a los coordinadores de las siete regiones sanitarias del municipio para la difusión e inicio de la investigación. Para estandarizar el proceso, la investigadora principal entregó personalmente un sobre que contenía el instrumento de recolección de datos, instrucciones para su cumplimentación y devolución, así como dos copias del Formulario de Consentimiento Libre e Informado (FCLI). En ese momento, se proporcionaron las mismas instrucciones verbalmente a todos los participantes, con el fin de reducir posibles sesgos en la recolección de datos.

El cuestionario completado, así como el FCLI firmado, debía colocarse en el mismo sobre sellado y entregarse a la administración del centro de salud, a la que se le indicó que lo remitiera a la coordinación regional de salud correspondiente para su posterior recogida por la investigadora. No obstante, fueron necesarios contactos periódicos con los centros de salud y los profesionales para recordarles la importancia de participar en el estudio y completar el cuestionario. De ese modo, la recolección de cuestionarios se realizaba mensualmente, tanto en las oficinas de coordinación regional como directamente en los centros de salud.

Algunos participantes solicitaron un nuevo sobre con el instrumento debido a la pérdida del material o porque se incorporaron al equipo después de que comenzara la recopilación de datos. Esta situación se debió principalmente a un proceso de contratación pública que supuso la sustitución de profesionales contratados temporalmente por personal fijo, así como a la pandemia de COVID-19, que obligó a reorganizar la atención y a reasignar las actividades de los profesionales entre los distintos servicios.

### Procesamiento y análisis de datos

Las respuestas a los cuestionarios se introdujeron por duplicado en una hoja de cálculo de Microsoft Excel 2013 para identificar posibles inconsistencias y se analizaron con el *software* estadístico Jamovi^®^, adoptando un nivel de significancia del 5%. Se utilizó la prueba de chi-cuadrado para verificar la asociación entre la capacitación de los enfermeros y el conocimiento sobre la vigilancia de la mortalidad materna. Para identificar los factores asociados con la realización de investigaciones de mortalidad materna (resultado), se realizó una regresión logística binaria, estimando *odds ratios* (OR) con intervalos de confianza del 95% (IC 95%)^20^.

Las variables se prepararon mediante codificación y categorización, y algunas variables se recodificaron para adaptarlas a la prueba utilizada: edad en rangos de edad (18-30, 31-40, 41-50, 51-60 y más de 61 años); tiempo de formación y tiempo de trabajo dicotomizados a partir de la mediana, con un valor de seis años (menor que/mayor o igual que la mediana); formación complementaria (sí/no) y nivel (pregrado/posgrado); y la forma del primer contacto con la familia para la investigación (presencial - ACS y visita domiciliaria/no presencial - teléfono y otros).

Por lo tanto, respetando la ausencia de multicolinealidad (examinada por el *variance inflation factor* y por tolerancia), todas las variables independientes se incluyeron en el modelo inicial, que posteriormente se refinó utilizando el método *backward stepwise* para obtener el modelo final. La bondad de ajuste se evaluó mediante estadísticas globales (*deviance y* criterios de información de Akaike y bayesianos), pseudo-R² (Cox & Snell y Nagelkerke) y prueba de razón de verosimilitud^21^.

Además, para evaluar su robustez y capacidad discriminativa, se realizó un análisis de rendimiento predictivo del modelo de regresión logística final. Se estimaron las métricas de precisión general, sensibilidad, especificidad, área bajo la curva ROC (AUC) y puntuación de Brier, lo que permitió examinar la estabilidad del modelo, complementando la evaluación del ajuste general, dada la naturaleza organizativa y multifactorial del resultado^19^.

### Aspectos éticos

El estudio fue aprobado por el Comité de Ética de la Investigación con Sujetos Humanos de la institución firmante, de acuerdo con la CAAE 22855519.1.0000.0021.

## Resultados

De los 143 enfermeros que participaron en el estudio, la mayoría eran del sexo femenino (n=130; 90,9%), tenían entre 18 y 60 años, con menos de seis años completos de experiencia en la profesión (n=101; 71,6%). En cuanto a su unidad de trabajo, el 74,8% estaban asignados a USF (n=107), el 17,5% a UBS (n=25) y el 7,7% a CF (n=11). En cuanto a su formación, el 65,8% (n=94) tenía hasta 10 años de experiencia desde su graduación en enfermería, y el 79,6% (n=110) había completado formación de posgrado, predominantemente a nivel *lato sensu* (n=103; 72%).

En cuanto a la investigación de muertes maternas, el 58,7% (n=84) informó haber realizado ya al menos una investigación, sin observarse diferencias estadísticamente significativas en cuanto al tipo de unidad en la que trabajan y la participación en la capacitación (p=0,396), la realización de la investigación de muertes maternas (p=0,942) o en cuanto a la discusión de casos en equipo (p=0,297). La principal fuente de información sobre el fallecimiento fue el agente comunitario de salud, ACS (n=34; 41,5%), seguido de los técnicos de vigilancia sanitaria (n=24; 29,3%) y los familiares (n=18; 22%).

La mayoría de los enfermeros no eran responsables de la atención prenatal de la mujer embarazada fallecida (n=59; 70,2%). La forma más común de contacto inicial con la familia para la investigación fue una visita domiciliaria (n=42; 52,5%), frecuentemente acompañada por otro profesional del equipo (n=62; 76,5%). En cuanto al soporte institucional, el 53,6% (n=45) informó haber recibido apoyo del equipo de vigilancia, mientras que el 46,4% (n=39) indicó falta de apoyo.

El 11% de los participantes reportó dificultades para completar los formularios de investigación, y solo un enfermero (1,2%) informó haber recibido recomendaciones del comité de mortalidad materna tras analizar el fallecimiento. El 32,9% (n=27) de los profesionales mencionó la discusión de los casos en reuniones de equipo, y el 30,5% (n=25) informó la negativa de la familia a participar en la investigación domiciliaria.

Si bien el 34,3% (n=49) de los enfermeros había participado en una capacitación específica sobre vigilancia de la mortalidad materna, la mayoría informó conocer el objetivo de la investigación (n=121; 84,6%), así como la existencia del comité de mortalidad materna en el municipio (n=100; 65,9%) y su objetivo (n=100; 69,9%). Sin embargo, solo el 17,5% (n=25) participó en las reuniones del comité.

La variable tiempo en el cargo mostró una asociación significativa con la participación en la capacitación sobre vigilancia de muertes maternas (p<0,001), con el conocimiento sobre la existencia del comité de mortalidad materna (p<0,001) y con la participación en una reunión del comité de mortalidad materna (p=0,005) (Tabla 2).

**Tabla 2:** Asociación entre variables del estudio y tiempo en el cargo actual (n* = 143). Campo Grande, MS, Brasil, 2019-2021

Variables	Tiempo en el cargo actual (APS^†^)		
	<6 años	6 años o más	Valor de p^‡^
	n* (%)^§^	n* (%)^§^	
Capacitación en vigilancia de la mortalidad materna			
Sí	21 (20,8)	28 (70)	<0,001
No	80 (79,2)	12 (30)	
Conocimiento sobre el comité de mortalidad materna del municipio			
Sí	62 (62,6)	37 (92,5)	<0,001
No	37 (37,4)	3 (7,5)	
Conocimiento del propósito del comité de mortalidad materna en el municipio			
Sí	64 (66)	35 (89,7)	0,005
No	9 (29,0)	24 (36,4)	
Participación en la reunión del comité			
Sí	4 (12,1)	13 (32,5)	0,004
No	89 (88.1)	27 (67,5)	
Conocimiento sobre el objetivo de la investigación de muertes maternas			
Sí	84 (85,7)	36 (92,3)	0,291
No	14 (14.3)	3 (7,7)	

*n = tamaño de la muestra compuesta por enfermeros; ^†^APS = Atención Primaria de Salud; ^‡^Prueba de chi-cuadrado, considerando un nivel de significancia del 5%; ^§^% = Porcentaje

La participación en la capacitación sobre vigilancia de muertes maternas mostró asociaciones significativas con variables relacionadas con el conocimiento sobre el objetivo de la investigación de la muerte (p=0,025), la existencia del comité de mortalidad materna (p<0,001) y su propósito (p<0,001), así como la participación en cualquier reunión del comité (p=0,003), como se muestra en la Tabla 3.

**Tabla 3:** Asociación entre variables del estudio y capacitación en vigilancia de mortalidad materna (n* = 143). Campo Grande, MS, Brasil, 2019-2021

Variables	Capacitación en vigilancia de la mortalidad materna		
	Sí	No	Valor de p^‡^
	n*(%^†^)	n*(%^†^)	
Conocimiento sobre el comité de mortalidad materna del municipio			
Sí	46 (93,9)	54 (58,7)	<0,001
No	3 (6,1)	38 (41,3)	
Conocimiento sobre el propósito del comité de mortalidad materna en el municipio			
Sí	45 (93,8)	55 (61,1)	<0,001
No	3 (6,3)	35 (38,9)	
Participación en reuniones del comité			
Sí	15 (30,6)	10 (10,6)	0,003
No	34 (69,4)	84 (89,4)	
Conocimiento sobre el objetivo de la investigación de muertes maternas			
Sí	46 (95,8)	75 (82,4)	0,025
No	2 (4,2)	16 (17,6)	
Dificultad para completar el Formulario de Investigación de Muerte Materna - M1^§^			
Sí	2 (5.0)	7 (16,7)	0,091
No	38 (95,0)	35 (83,3)	
Dificultad para completar el Formulario de Investigación de Muerte Materna - M3^||^			
Sí	3 (7,5)	6 (14,3)	0,326
No	37 (92,5)	36 (85,7)	

*n = Tamaño de la muestra compuesta por enfermeros; ^†^% = Porcentaje; ^‡^Prueba de Chi-cuadrado, considerando un nivel de significancia del 5%; ^§^M1 = Formulario de Investigación de Muerte Materna - Servicio de Salud Ambulatorio; ^||^M3 = Formulario de Investigación de Muerte Materna - Entrevista domiciliaria

A su vez, el conocimiento sobre la existencia del comité de mortalidad materna se asoció significativamente con el conocimiento sobre el propósito del comité, la participación en las reuniones y un mayor conocimiento sobre el objetivo de la investigación de muertes maternas (Tabla 4).

**Tabla 4:** Asociación entre las variables del estudio y el conocimiento sobre la existencia del comité de mortalidad materna en el municipio (n* = 143).Campo Grande, MS, Brasil,2019-2021

Variables	Conocimiento sobre el comité de mortalidad materna del municipio		
	Sí	No	Valor de p^‡^
	n* (%^†^)	n* (%^†^)	
Conocimiento del propósito del comité de mortalidad materna en el municipio			
Sí	84 (87,5)	14 (35,0)	<0,001
No	12 (12,5)	26 (65,0)	
Participación en reuniones del comité			
Sí	24 (24,0)	1 (2,4)	0,002
No	76 (76,0)	40 (97,6)	
Conocimiento sobre el objetivo de la investigación de muertes maternas			
Sí	90 (92,8)	29 (72,5)	0,001
No	7 (7,2)	11 (27,5)	

*n = Tamaño de la muestra compuesta por enfermeros; ^†^% = Porcentaje; ^‡^Prueba de Chi-cuadrado, considerando un nivel de significancia del 5%

La Tabla 5 presenta los coeficientes del modelo final de regresión logística binaria. La intersección no fue estadísticamente significativa (β=-0,128; SE=0,207; Z=-0,618; p=0,536). En cambio, la participación en la capacitación se asoció fuertemente con la realización de investigaciones de muertes maternas, con un coeficiente positivo significativo (β=1,762; SE=0,438; Z=4,020; p<0,001). Esta estimación corresponde a una razón de probabilidades de 5,82 (IC 95%: 2,46-13,75), lo que indica que los profesionales que participaron en la capacitación tuvieron casi seis veces más probabilidades de haber realizado una investigación en comparación con aquellos que no recibieron capacitación.

**Tabla 5:** Coeficientes del modelo final de regresión logística binaria, entre variables profesionales y operativas y la realización previa de investigación de muertes maternas por parte de enfermeros de Atención Primaria de Salud. Campo Grande, MS, Brasil, 2019-2021

Predictores	β*	SE^†^	Z^‡^	p^§^	OR^||^ (IC 95%^¶^)
Intercepto	-0,128	0,207	-0,618	0,536	0,88 (0,58 - 1,32)
Capacitación en vigilancia de la mortalidad materna					
Sí - No	1,762	0,438	4,020	<0,001	5,82 (2,46 - 13,75)
*Deviance* = 174; AIC^**^ = 178; R²CS^††^ = 0,128; R²N^‡‡^ = 0,173; VIF^§§^ = 1,00; Tolerancia = 1,00					

*β = Coeficiente estimado en el modelo logístico (logit); ^†^SE = Error estándar de la estimación; ^‡^Z = Estadística de prueba de Wald; ^§^p = Nivel de significancia; ^||^OR = *Odds Ratio* (Razón de probabilidades); ^*¶*^ IC 95**%** = Intervalo de confianza del 95%; **AIC = Criterio de Información de Akaike; ^††^R²CS = Coeficiente de determinación de Cox & Snell; ^‡‡^R²N = Coeficiente de determinación de Nagelkerke; ^§§^VIF = *Variance Inflation Factor* (Factor de inflación de la varianza)

El modelo de regresión logística presentó un ajuste global satisfactorio, como lo demuestran el valor de la atenuación, igual a 174, y el Criterio de Información de Akaike (AIC=178), lo que sugiere un buen equilibrio entre complejidad y capacidad predictiva. Las medidas de pseudo-R² indicaron un rendimiento moderado del modelo, con un R² de Cox & Snell de 0,128 y un R² de Nagelkerke corregido de 0,173. La evaluación de los supuestos del modelo no reveló colinealidad, ya que el Factor de Inflación de la Varianza (VIF=1,00) y la tolerancia (1,00) indicaron una independencia entre el predictor incluido y ausencia de redundancia estructural que pudiera comprometer la estabilidad de las estimaciones.

El análisis del rendimiento predictivo indicó una precisión del 63,6%, con un área bajo la curva ROC (AUC) de 0,672, lo que sugiere una capacidad discriminatoria moderada. El modelo mostró una alta especificidad (86,2%) y una sensibilidad moderada (48,2%), lo que indica una mayor capacidad para identificar correctamente a los profesionales que no habían realizado la investigación de la muerte materna. El índice de Brier fue de 0,210, lo que pone de manifiesto un error de calibración aceptable y la estabilidad de las probabilidades estimadas.

## Discusión

Los resultados de este estudio indicaron que el desempeño de los enfermeros de la APS en la vigilancia de la mortalidad materna está directamente relacionado con la participación en la capacitación específica sobre el tema, constituyendo el único factor significativamente relacionado con el desempeño previo de las investigaciones de mortalidad materna. La magnitud de esta asociación, con una probabilidad casi seis veces mayor entre los profesionales capacitados, refuerza la centralidad de la educación continua como elemento estructurador para fortalecer la vigilancia de la salud y mejorar las prácticas de investigación en el ámbito de la APS^22^.

Aunque la mayoría de los enfermeros participantes poseen En la formación complementaria de posgrado (*lato sensu)*, una baja proporción de profesionales participó en la formación específica sobre vigilancia de la mortalidad materna. Además, la ausencia de diferencias entre los tipos de unidades de trabajo indica que las deficiencias relacionadas con la discusión de casos y la investigación de la mortalidad materna no se limitan a un modelo de servicio específico, sino que reflejan desafíos organizativos transversales a la atención primaria.

Este hallazgo sugiere que la formación académica general, incluso si es avanzada, no es suficiente para garantizar el desarrollo de las habilidades técnicas, analíticas y operativas necesarias para la adecuada realización de investigaciones de muertes, corroborando la evidencia de que la vigilancia de la mortalidad materna exige conocimientos específicos y actualizados alineados con las directrices nacionales e internacionales^23^.

La formación continua, en este contexto, contribuye a la mejora de la autonomía técnica y la seguridad profesional, además de favorecer el acceso a directrices nacionales e internacionales y la incorporación de prácticas alineadas con las políticas públicas destinadas a reducir la mortalidad materna. Si bien la ausencia de formación no indica una falta de conocimiento de los principios de vigilancia de la mortalidad materna, los resultados sugieren que la pericia necesaria para realizar investigaciones no se construye exclusivamente con experiencia práctica o formación académica, sino que requiere inserción en procesos de formación específicos, orientados a la vigilancia y la cualificación de la atención^22^ ^)-(^ ^23^.

La participación en la capacitación se asoció con mayor antigüedad en la APS, conocimiento sobre la existencia y los objetivos del comité de mortalidad y participación en sus reuniones. Estos resultados indican que la antigüedad implica experiencia acumulada, lo que puede aumentar la exposición de los profesionales a la capacitación y a los procesos organizativos relacionados con la vigilancia y favorecer el desarrollo de competencias específicas. Sin embargo, los hallazgos demuestran que la antigüedad por sí sola no garantiza una participación efectiva en la vigilancia de la mortalidad materna, lo que refuerza la necesidad de estrategias institucionales sistemáticas para la formación continua.

La capacitación específica permite un contacto más cercano con las etapas esenciales del proceso de vigilancia de la mortalidad, lo que mejora la recopilación y obtención de información importante para el análisis crítico de casos por parte de otras instancias en la red, como el comité MM y el equipo de vigilancia de la salud^24^. La calidad de los datos registrados en cada punto de la red de atención y puestos a disposición por los sistemas de información sanitaria está directamente relacionada con la subnotificación y el subregistro^25^, lo que compromete el desarrollo de recomendaciones y el potencial preventivo de la vigilancia.

El modelo de regresión logística mostró un rendimiento explicativo moderado, como lo indican los valores R² de Cox & Snell y Nagelkerke, lo que refleja la naturaleza multifactorial y organizativa de la vigilancia de la mortalidad materna y destaca que, si bien la formación profesional es un factor central, existen otros determinantes relevantes no incluidos en las variables analizadas.

Los factores institucionales, organizativos y contextuales, como la disponibilidad de recursos humanos, el apoyo institucional, la rotación de personal, la carga de trabajo, la cultura organizacional y las condiciones de salud mental de los trabajadores, pueden influir significativamente en la realización de las investigaciones y deben ser considerados en estudios futuros. Por lo tanto, aunque la capacitación se ha destacado como un factor central, no debe entenderse de forma aislada, sino integrada en un conjunto más amplio de intervenciones sistémicas^23^.

Entre los hallazgos operativos, se destaca el rol del Agente Comunitario de Salud (ACS) como fuente primaria de información sobre la muerte materna, así como el uso de visitas domiciliarias como estrategia predominante para el contacto inicial con la familia, frecuentemente llevadas a cabo con la participación de más de un profesional del equipo. Estas prácticas se alinean con los principios de la APS y de la Vigilancia de la Salud, especialmente en lo que se refiere al vínculo previo con la familia de la mujer embarazada, la identificación temprana de eventos y el enfoque interprofesional^18^ ^),(^ ^26^. El ACS, debido a su participación comunitaria y vínculo previo con las familias, cumple un rol estratégico en la detección y notificación de muertes, lo que refuerza la importancia de su capacitación y articulación con los enfermeros, quienes actúan como coordinadores del equipo^27^.

La Atención Primaria de Salud puede instrumentarse mediante diferentes modelos de atención y arreglos organizativos siempre que se preserven sus atributos esenciales, como el primer contacto, la longitudinalidad, la integralidad y la coordinación de la atención^25^. En el municipio estudiado, aunque la APS está compuesta por unidades de salud de diferentes tipos (USF, UBS y CF), todas son responsables de investigar las muertes maternas en el territorio asignado, lo que corrobora el resultado de este estudio que no indicó diferencias estadísticamente significativas con respecto al tipo de unidad de acción y participación en la capacitación, la realización de investigaciones de muertes maternas y la discusión de casos en equipo.

A pesar de estas potencialidades, el estudio reveló debilidades operativas críticas en el proceso de vigilancia de la mortalidad materna. El hecho de que solo un enfermero recibiera recomendaciones del comité de mortalidad materna tras analizar el caso resulta particularmente preocupante, ya que compromete el carácter pedagógico, preventivo y transformador de la vigilancia. La ausencia de devoluciones sistemáticas debilita el ciclo de retroalimentación dentro de los servicios, limita la incorporación del aprendizaje institucional y reduce el potencial de las recomendaciones para reorganizar los procesos de trabajo y mejorar la calidad de la atención^24^ ^)-(^ ^25^.

De igual modo, la baja proporción de profesionales que informaron haber discutido casos de muerte materna en reuniones de equipo resalta la debilidad de los espacios colectivos para la reflexión y el análisis crítico de la atención. La ausencia de estas discusiones dificulta la identificación de fallas de atención evitables, compromete la toma de decisiones compartida y debilita el aprendizaje organizacional^28^.

A nivel internacional, factores como la falta de tiempo, liderazgo y soporte institucional, la comunicación limitada, los roles profesionales poco claros y los recursos restringidos también tienden a obstaculizar la colaboración interprofesional en los servicios de salud^29^ ^)-(^ ^32^. Este escenario puede estar relacionado con la sobrecarga de trabajo, la falta de tiempo protegido para reuniones, la insuficiente valoración de estas prácticas en la rutina diaria de la Atención Primaria de Salud y la fragilidad de una cultura organizacional centrada en el análisis de eventos adversos^33^.

Además, la sobrecarga de cuidados y el desgaste físico y emocional de los profesionales, intensificados en el contexto de la pandemia, también deben considerarse como factores que pueden haber influido negativamente en la participación en actividades de vigilancia, formación continua y debates en equipo. El *Burnout* y el sufrimiento psicológico entre los trabajadores sanitarios se han descripto ampliamente en la literatura como elementos que afectan la calidad de la atención y el cumplimiento de las prácticas institucionales, por lo que también deben incorporarse a los análisis de vigilancia de la mortalidad materna^25^ ^),(^ ^34^ ^)-(^ ^35^.

Otro hallazgo relevante se relaciona con el soporte institucional, ya que casi la mitad de los enfermeros del estudio informaron no recibir apoyo del equipo de vigilancia sanitaria. Esta brecha pone de manifiesto las debilidades en la articulación entre la APS y los demás componentes del sistema de vigilancia, lo que puede generar inseguridad profesional, dificultades operativas y discontinuidad en las acciones de investigación. El apoyo técnico e institucional es fundamental para garantizar la calidad de las investigaciones, especialmente en contextos de mayor vulnerabilidad social y complejidad asistencial^25^.

La ausencia de seguimiento prenatal por parte de una porción significativa de los enfermeros a cargo de la investigación de la muerte materna es un factor digno de mención. La atención prenatal es crucial para la detección temprana de riesgos obstétricos, la implementación de intervenciones oportunas y el fortalecimiento del vínculo con la mujer embarazada. Las fallas en este seguimiento pueden reflejar problemas organizativos, limitaciones de acceso, recursos insuficientes, así como factores sociales y culturales que influyen en la adherencia de las mujeres a los servicios^36^ ^)-(^ ^37^. La resistencia o negativa de la familia a participar en la investigación domiciliaria puede estar relacionada con la percepción de la calidad de la atención recibida y la trayectoria de la mujer dentro de la red de atención médica.

Sin embargo, estos resultados deben interpretarse considerando el inicio de la pandemia de COVID-19 en el contexto brasileño. Este evento, incontrolable durante la planificación del estudio, afectó prácticamente todo el proceso de recolección de datos y provocó transformaciones en el funcionamiento de la APS. La reorganización de los servicios, la priorización de las acciones de emergencia, la suspensión o reducción de la atención colectiva y de actividades organizativas, así como la limitación de las visitas domiciliarias, entre otros cambios, pudieron haber contribuido a la baja adherencia a las etapas del proceso de vigilancia observada en el estudio, especialmente en los procesos de formación continua, discusión en equipo y retroalimentación institucional de los análisis de los comités.

También debe considerarse el contexto de la pandemia en relación con las limitaciones de este estudio. La tasa de no respuesta de aproximadamente el 30% de los profesionales elegibles, posiblemente relacionada con la sobrecarga asistencial durante este período, asociada al muestreo por conveniencia y la falta de información sobre quienes no respondieron, puede introducir un sesgo de respuesta, ya que los profesionales con mayor sobrecarga o menor participación en la vigilancia epidemiológica podrían haber sido menos propensos a participar, lo que podría sobreestimar el nivel de conocimiento y participación observado.

Estas limitaciones, sumadas al hecho de que el estudio se realizó en un solo municipio y al poder explicativo moderado del modelo, indican la necesidad de futuras investigaciones que incorporen variables organizativas, institucionales y psicosociales, así como análisis estratificados por tipo de unidad de salud, teniendo en cuenta las especificidades de los diferentes modelos de organización de la APS.

A pesar de estas limitaciones, el estudio logró incluir a profesionales de todas las regiones sanitarias del municipio y contó con un número de participantes que superó el tamaño mínimo de muestra requerido para garantizar la solidez del análisis. Los hallazgos, aunque no representativos debido a sesgos en la selección de la muestra, destacan de manera consistente^18^ factores modificables que pueden apoyar a los gestores, de diferentes contextos, en la formulación de políticas institucionales dirigidas a fortalecer la formación continua en salud, la articulación intersectorial y el apoyo institucional a los equipos. Asimismo, presenta posibles líneas de investigación para futuros estudios.

Por lo tanto, este estudio proporciona información importante e inédita no solo para la práctica de enfermería en la vigilancia de la mortalidad materna, sino que además demuestra la necesidad de aplicar un enfoque integrado, que se extienda más allá de la cualificación individual e incorpore estrategias para fortalecer la gestión, el trabajo interprofesional, la cultura organizacional y las condiciones laborales, a fin de contribuir a la disminución de la mortalidad materna y la mejora de la atención sanitaria de las mujeres. Un sistema de salud fortalecido, con profesionales capacitados y que garantice la atención sanitaria de las mujeres en sus diferentes ciclos de vida, especialmente en el *continuum* del embarazo, el parto, el posparto y el período perinatal, es fundamental para la reducción de la mortalidad materna y neonatal^24^ ^),(^ ^38^.

## Conclusión

Las variables relacionadas con la capacitación en vigilancia de la mortalidad materna resultaron esenciales para el desempeño del personal de enfermería en la Atención Primaria de Salud (APS), lo que refuerza la relevancia de la formación continua para el desarrollo de esta práctica. El estudio también identificó la necesidad de ampliar los espacios de discusión de casos de mortalidad materna entre los equipos, así como de garantizar el retorno sistemático de los análisis de los comités de mortalidad materna a la APS, con el fin de mejorar las investigaciones y optimizar el proceso de trabajo.

La evidencia obtenida apunta al potencial para orientar ajustes en los procesos de trabajo en equipo y las acciones intersectoriales, respaldar las políticas institucionales de formación continua y fortalecer la asistencia. Estos elementos contribuyen a la mejora y la calidad de la vigilancia sanitaria, en consonancia con el cumplimiento de los objetivos internacionales establecidos, como la meta 3.1 de los Objetivos de Desarrollo Sostenible (ODS) para la reducción de la tasa de mortalidad materna (TMM).

## Data Availability

Todos los datos generados o analizados durante este estudio están incluidos en este artículo publicado.
